# An integrative approach using real-world data to identify alternative therapeutic uses of existing drugs

**DOI:** 10.1371/journal.pone.0204648

**Published:** 2018-10-09

**Authors:** Kouichi Hosomi, Mai Fujimoto, Kazutaka Ushio, Lili Mao, Juran Kato, Mitsutaka Takada

**Affiliations:** 1 Division of Clinical Drug Informatics, School of Pharmacy, Kindai University, Kowakae, Higashi-osaka, Osaka, Japan; 2 Innovation and Entrepreneurship, Research, Takeda Pharmaceutical Company Limited, Muraoka-Higashi, 2- Chome, Fujisawa, Kanagawa, Japan; National Chiao Tung University College of Biological Science and Technology, TAIWAN

## Abstract

Different computational approaches are employed to efficiently identify novel repositioning possibilities utilizing different sources of information and algorithms. It is critical to propose high-valued candidate-repositioning possibilities before conducting lengthy *in vivo* validation studies that consume significant resources. Here we report a novel multi-methodological approach to identify opportunities for drug repositioning. We performed analyses of real-world data (RWD) acquired from the United States Food and Drug Administration’s Adverse Event Reporting System (FAERS) and the claims database maintained by the Japan Medical Data Center (JMDC). These analyses were followed by cross-validation through bioinformatics analyses of gene expression data. Inverse associations revealed using disproportionality analysis (DPA) and sequence symmetry analysis (SSA) were used to detect potential drug-repositioning signals. To evaluate the validity of the approach, we conducted a feasibility study to identify marketed drugs with the potential for treating inflammatory bowel disease (IBD). Primary analyses of the FAERS and JMDC claims databases identified psycholeptics such as haloperidol, diazepam, and hydroxyzine as candidates that may improve the treatment of IBD. To further investigate the mechanistic relevance between hit compounds and disease pathology, we conducted bioinformatics analyses of the associations of the gene expression profiles of these compounds with disease. We identified common biological features among genes differentially expressed with or without compound treatment as well as disease-perturbation data available from open sources, which strengthened the mechanistic rationale of our initial findings. We further identified pathways such as cytokine signaling that are influenced by these drugs. These pathways are relevant to pathologies and can serve as alternative targets of therapy. Integrative analysis of RWD such as those available from adverse-event databases, claims databases, and transcriptome analyses represent an effective approach that adds value to efficiently identifying potential novel therapeutic opportunities.

## Introduction

Real-world data (RWD) is defined as data derived from sources associated with outcomes of a patient population in real-world settings, which include medical records, claims databases, prescription databases, and spontaneous adverse drug-event reports databases. RWD has attracted attention, particularly in pharmacovigilance and epidemiological studies. Various analytical methods have been developed to translate RWD to a hypothesis or an idea such as unexpected associations between drugs and adverse events (AEs) [1–5]. Among all methods for analysis of AEs, sequence symmetry analysis (SSA) and disproportionality analysis (DPA) are known for their moderate sensitivity and high specificity [6], and these methods have been used as complementary tool in pharmacovigilance. SSA, which evaluates prescriptions or claims databases, and DPA, which evaluates spontaneous adverse drug-event reports databases, are frequently used to predict potential risks associated with drugs. SSA and DPA are computationally fast approaches that employ simple algorithms and therefore serve as useful tools for efforts to conduct global pharmacovigilance of medicine. In addition, combined use of DPA and SSA can enhance signal detection, because SSA method detects an additional true positive signal that were not detected by DPA algorithms alone [6]. We reported studies using these methodologies and algorithms to detect signals of AEs. Inverse associations generally detected in using SSA and DPA have no meaning and are thus commonly disregarded. However, we noted that inverse associations between target drugs and AEs are often present, which suggest potential alternative therapeutic opportunities [7]. To our knowledge, there are several reports on evaluation of these inverse associations for their benefits to other methodologies such as drug-repositioning approaches [8, 9]. This study adds another layer to the approach by employing the analysis of dysregulated biogroups, which was not done in the other studies.

The value of RWD is also increasingly recognized to uncover alternative use of approved drugs [10]. It is the trend now to connect the drug candidate and their potential new applications by data mining of RWD to build the network of pharmacological elements [11]. DPA has proven useful in finding unknown drug effects [7]. Recently, we reported that integrative approach using SSA and DPA showed inverse association between sodium channel-blocking antiepileptic drug use and several cancers [8]. A number of experimental studies suggest that sodium channel-blocking antiepileptic drugs are potential candidates for anti-cancer agents. Although SSA and DPA have been originally developed as complementary tool in pharmacovigilance, we hypothesized that these methods can be a powerful tool to explore its unknown clinical effect.

Here we developed a novel multi-methodological approach to assess the value of these inverse associations and to determine whether they suggest novel pharmacological effects of approved drugs. These methodologies may serve as an alternative, robust tool for drug repositioning, because they complement conventional approaches that generate drug-repositioning possibilities by comparing patterns of drug-associated AEs [2, 3].

The standard methods employed for drug discovery are costly, time-consuming, and burdened by a very low success rate. The pharmaceutical industry is therefore facing major challenges to its attempts to increase the productivity of research and development (R&D) [12, 13]. To address this issue, major efforts have been made to repurpose approved drugs to reduce the cost and duration of R&D and to minimize the risk of AEs [14]. A computational approach is particularly appealing because of the ability to rapidly screen candidates and to reduce the number of possible repositioning candidates in an unbiased manner [1, 15, 16]. For successful drug discovery, it is critical to take an approach comprising traditional target-based drug discovery and forward chemical genetics, including phenotype analysis [17]. Further, OMICs data are considered a valuable source of phenotypic information, and genome-wide association studies, transcriptomics, epigenomics, proteomics, and metabolomics data are publicly available. Transcriptome data constitute the largest collection[18] and are therefore widely used for drug repositioning [15, 19–22].

The purpose of the present study was to develop and evaluate the validity of a multi-methodological approach for sequentially analyzing RWD for claims and spontaneous adverse events, followed by gene expression profiling to generate drug-repositioning possibilities. As a demonstration of the approach, we performed analysis to answer the question whether any marketed drug can be repositioned for GI diseases. First, we analyzed the drugs for respiratory disease and antipsychotic drugs. As a result, inverse associations were detected between use of antipsychotic drugs and GI diseases, but not between use of drugs for respiratory disease and GI diseases. Therefore, we focused on psycholeptics in current study. Here we report the association between psycholeptic use and decreased diagnosis of inflammatory bowel disease (IBD) (Crohn's disease [CD] and ulcerative colitis [UC]) newly identified from integrative analyses of claims databases and spontaneous AE-reports databases as well as transcriptome analyses. We also discuss the potential application of our findings related to drug repositioning. In SSA, when significant inverse associations were detected at least three intervals among 6-, 12-, 24-, 36-months intervals, we defined such associations as inverse signal. In DPA, when the reporting odds ratio (ROR) and information component (IC) met the criteria, we defined such associations as inverse signal. Additionally, when inverse signals were confirmed by SSA and DPA, we defined such inverse signals as drug-repositioning signal.

## Materials and methods

**Fig 1:**
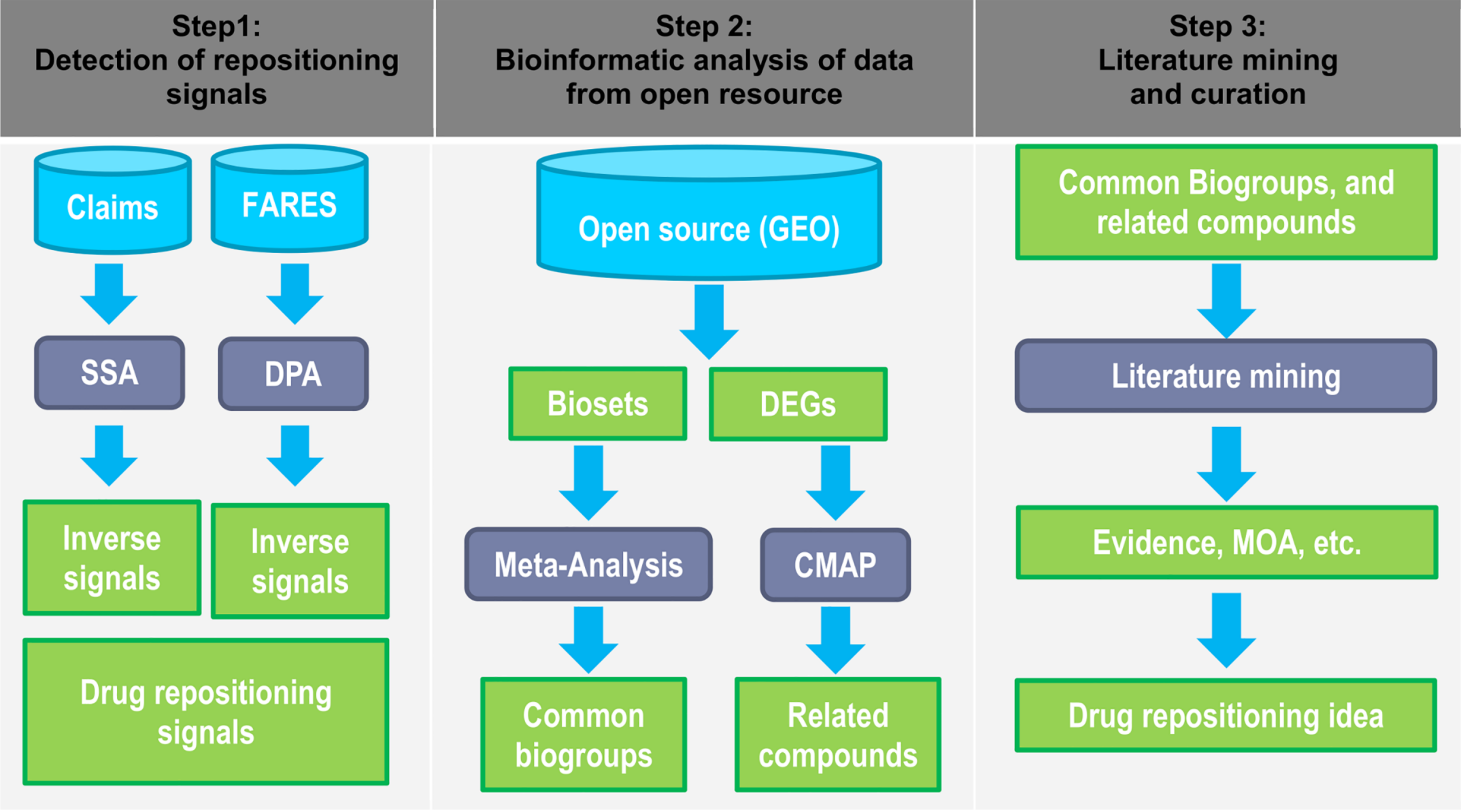
Work flow of the multi-methodological approach. Step 1, sequence symmetry analysis (SSA) of prescriptions or claims database and disproportionality analysis (DPA) of spontaneous adverse drug-event reports database to detect inverse signals and drug-repositioning signal. An inverse signal indicates an inverse association between the number of prescription drugs and the diagnosis of the associated diseases. The drug-repositioning signal indicates the therapeutic benefit revealed by the inverse signal identified using SSA and DPA; Step 2, bioinformatics analysis of data from open sources to cross-validate the drug-repositioning signal. Gene expression data for diseases and their related drugs, extracted from open sources (Gene Expression Omnibus, GEO) as a bioset containing differentially expressed genes (DEGs). Meta-analysis discovered commonly perturbed biogroups (i.e., pathways) among biosets. Connectivity MAP (CMAP) analysis identified related compounds with similar gene signatures as extracted biosets; Step 3, literature mining and curation to generate drug-repositioning possibilities. Common biogroups and associated compounds identified in step 2 were profiled by literature mining for supportive evidence and the underlying mechanism of action (MOA) to generate drug-repositioning signals.

The workflow of the study is summarized in Fig 1. First, data mining of real-world databases, including claims and spontaneous AE databases, was performed to identify an inverse association between prescription drugs and the diagnosis of IBD (CD and UC). These data mining methods were developed for detecting safety signals for application to pharmacovigilance. SSA has moderate sensitivity and high specificity for detecting safety signals of drugs [23]. SSA was also reported as a complementary pharmacovigilance tool for DPA to detect safety signals [24]. There have been studies using integrative approaches of SSA and DPA to find safety signals [25, 26]. In addition, DPA has been utilized to find unknown drug effects, for which several studies on drug repositioning have been reported [7]. In our study, an integrative approach using SSA and DPA was applied to identify repositioning opportunities for marketed drugs.

### Sequence symmetry analysis

#### Data sources

A large and chronologically organized claims database constructed by the Japan Medical Data Center Co., Ltd (JMDC, Tokyo, Japan) [27] was used in the present study. The JMDC claims database includes prescription data for approximately 3.75 million insured persons (approximately 3.1% of the population) comprising mainly company employees and their family members. All Japanese citizens are required to be enrolled in one of three types of health insurances: occupation-based, municipality-based, or a separate system for elder people over 75 years old. All insured medical and pharmaceutical institutions issue medical and pharmacy claims for each patient every month to ensure reimbursement for the costs of health-related service. Because most beneficiaries are working adults or their family members, the proportion of elderly patients aged ≥65 years is low. The JMDC claims database collects monthly claims from medical institutions and pharmacies and provides information on the beneficiaries, including encrypted personal identifiers, age, and sex. The JMDC claims database also contains information of International Classification of Diseases 10^th^ revision (ICD-10) procedure and diagnostic codes as well as the name, dose, and number of days supplied with the prescribed or dispensed drugs, or both. Drugs were coded according to the Anatomical Therapeutic Chemical classification of the European Pharmaceutical Market Research Association (EPHMRA). An encrypted personal identifier was used to link claims data from different hospitals, clinics, and pharmacies. For the SSA, we utilized cases extracted from the JMDC claims database that includes psycholeptics prescribed at least once during the study period, accompanied by a diagnosis of CD or UC.

The Ethics Committee of Kindai University School of Pharmacy approved this study. The names and identification numbers of the JMDC claims database were replaced by a univocal numeric code, rendering the database anonymous at the source. Therefore, subjects’ informed consent was not required.

#### Definition of psycholeptics, CD, and UC

EPHMRA Anatomical and Therapeutic Chemical codes N05A, N05B, and N05C were defined as psycholeptics in the present study. Combination products were not included in the analysis. The ICD-10 codes K50 and K51 were defined as CD and UC, respectively.

#### Data mining

SSA was performed to evaluate the associations between the use of psycholeptics and the diagnoses of CD or UC. The SSA method was originally developed to investigate the associations between the use of certain targeted drugs and potential AEs [28, 29]. In the present study, the inverse signal obtained from SSA was defined as a drug-repositioning signal. Briefly, SSA evaluates asymmetry in the distribution of an event before and after the initiation of a specific treatment. Asymmetry may indicate an association between the specific treatment of interest and the event. In the present study, we analyzed the inverse association between psycholeptic use and the diagnosis of CD and UC.

The crude sequence ratio (SR) was defined as the ratio of the numbers of patients newly diagnosed with CD or UC before and after the initiation of psycholeptics, respectively. SR is sensitive to changes in event (prescribing or diagnosis) trends over time. Event order is affected by trends in sales of the considered drugs and frequency of the considered diagnoses over a time-window. If an event occurred with increasing frequency, a non-specific excess of patients with subsequent secondary events would be expected; therefore, SRs were adjusted for temporal trends in events (psycholeptic prescribing and diagnosis of inflammatory bowel disease) using the method proposed by Halls [28]. The probability for a psycholeptic to be prescribed first, in the absence of any causal relationship, can be estimated from a null-effect SR. The null-effect SR produced by the proposed model may be interpreted as a reference value for the SR. Thus, the null-effect SR is the expected SR in the absence of any causal association, accounting for trends in event frequency. By dividing the crude SR by the null-effect SR, an adjusted sequence ratio (ASR) which is corrected for temporal trends can be obtained [28]. An ASR <1 indicates an inverse association of psycholeptic use with risks of CD and UC. We used a slightly modified model to account for the limited time between psycholeptic use and diagnoses of CD and UC [29].

All incident users of psycholeptics and all newly diagnosed patients with CD or UC were identified from January 2005 to March 2016. To exclude prevalent users of psycholeptics, the analysis was restricted to users whose first prescription was administered during July 2005 or later (after a run-in period of 6 months). Similarly, the analysis was restricted to patients whose first diagnosis was July 2005 or later. A waiting-time distribution analysis was performed to ensure that our analysis was restricted to incident users of psycholeptics and patients newly diagnosed with CD or UC [30]. An identical run-in period was applied to patients enrolled in the cohort after June 2005. We identified incident users by excluding patients who received their first psycholeptic prescription before July 2005. We identified patients newly diagnosed with CD or UC by excluding patients whose first diagnosis of CD or UC was earlier than July 2005. We identified patients who had initiated new treatment with psycholeptics and whose first diagnosis of CD or UC was within 6-, 12-, 24-, and 36-month periods (intervals) of the initiation of treatment. SSA had lower sensitivity when the time between events was restricted to a short period. This low sensitivity is possibly due to small sample size and inadequate time window frame particularly for drug effects that may take longer to manifest. When long interval was used, within subject bias, such as maturation and other potential time-varying covariates (e.g. change in diet) that could happen in a long study period, it would make it difficult to determine the causality of exposure and outcome. To date, a definite range of interval has not been established. In our study, when significant inverse associations were detected at least three intervals among 6-, 12-, 24-, 36-months intervals, we defined such associations as inverse signal.

### Disproportionality analysis

#### Data source

The FDA’s Adverse Event Reporting System (FAERS) is a computerized database designed to support the FDA’s after-market safety surveillance program for all approved drugs and therapeutic biologicals. The raw data from the FAERS database is available without charge from http://www.fda.gov/Drugs/InformationOnDrugs/ucm135151.htm. The present study included FAERS data from the first quarter of 2004 through the end of 2015, representing 7,561,254 reports. After excluding duplicate reports (common case numbers), we identified 86,139,835 drug-reaction pairs among 6,153,696 reports. The preferred terms (PTs) of the Medical Dictionary for Regulatory Activities (MedDRA version 19.0) were used to classify AEs.

#### Identifying psycholeptics, CD, and UC

An archive of drug names that included the names of all preparations, generic names, and synonyms of drugs marketed worldwide was created using the Martindale website (https://www.medicinescomplete.com/mc/login.htm). We identified the psycholeptics N05A, N05B, and N05C by linking this archive to the FAERS database. All records that included psycholeptics in the DRUG files were selected, and the relevant reactions from the REACTION files were then identified. AEs in the FAERS database were coded using MedDRA–PTs, which are grouped according to defined medical conditions or areas of interest. We used the Standardized MedDRA Queries to identify PTs related to CD and UC.

#### Data mining

DPA was originally developed to investigate the associations between the use of certain targeted drugs and potential AEs. In the present study, the inverse signal obtained from the DPA was defined as a drug-repositioning signal. The DPA is described in numerous reports. The reporting odds ratios (RORs) and information components (ICs) were utilized to detect signals. Signal scores were calculated using a case/noncase method [31, 32]. Those reports including the event of interest were defined as the cases, and the other reports comprised non-cases. By applying these algorithms and using a two-by-two table of frequency counts, we calculated signal scores to assess whether a drug was significantly associated with a diagnosis of CD or UC. In current study, drug-reaction pair-based count was used for analysis. However, these calculations or algorithms, so-called DPA, differ in that the ROR is frequentist (non-Bayesian) [33], whereas IC is Bayesian [34]. For the ROR, an inverse association was defined if the upper limit of the 95% two-sided confidence interval (95% CI) was <1. For the IC, an inverse association was defined if the upper limit of the 95% CI was <0. In the present study, two methods were used to detect inverse associations, and the associations between psycholeptic use and the diagnosis of CD or UC were listed as an inverse signal when the two indices met the criteria outlined above.

### Data management and statistical analysis

Data management and analysis of RWD were performed using Visual Mining Studio software version 8.1 (Mathematical Systems, Inc. Tokyo, Japan). The results of the analyses are expressed as mean ± standard deviation (SD) for quantitative data. The 95% CI of the ASR was calculated using a method to calculate exact CIs of binomial distributions [35].

### Meta-analysis of transcriptome data in NextBio

We searched the NextBio database (Now, BaseSpace Correlation Engine) (Illumina Inc., CA, USA) for curated gene expression profiles of the compounds and diseases of interest available from an open source: Gene Expression Omnibus (GEO). Compound names (haloperidol, diazepam, or hydroxyzine) and disease names (CD, or UC) were used as queries to filter gene expression datasets for subsequent gene expression profiling. For each query term, we identified one microarray dataset that met the inclusion criteria of differential mRNA expression data for humans acquired by analyses of a perturbed condition and unaffected control with a high signal-to-noise ratio. Details of the identified datasets are described in S12 Table. Gene expression datasets (i.e., biosets) were extracted from NextBio and subjected to analysis using the meta-analysis tool of NextBio, uses a normalized ranking approach to compare data from different studies, platforms, and methods [16]. Using meta-analysis, a correlation score was calculated for each concept (i.e., query compound or query disease) to indicate relevance to genes and biogroups. Genes and biogroups with p ≤0.05 indicated a significant association with a query compound or disease. A similar approach was used to identify a dysregulated gene as a potential biomarker for Parkinson Disease [36]. To profile the meta-analysis results from the present study, we performed literature mining and curated genes and biogroups, which were expressed at significantly higher levels in samples acquired from patients with IBD vs controls which are human-derived cell lines treated with haloperidol, diazepam, or hydroxyzine as well as those expressed at significantly lower levels compared with controls.

### Connectivity MAP (CMAP) analyses

After we identified the gene expression dataset for meta-analysis, differentially expressed genes (DEGs) were extracted for haloperidol, diazepam, or hydroxyzine. For diazepam and hydroxyzine, DEG probes with p ≤0.05 and an absolute fold-change ≥1.5 were included in the CMAP analyses [19, 37]. For haloperidol, the inclusion criterion was DEG probes with p ≥0.05 because of a small number of DEG probes if a fold-change cutoff was applied as well. These sets of probes were then filtered for compatibility with CMAP analysis (Broad Institute) [19]. When the reference signature for a drug matched our gene signature with p ≤0.05, it was shortlisted with the connectivity score to indicate its correlation with a query compound.

## Results

### Sequence symmetry analysis

The characteristics of the study population of SSA are summarized in S1 Table, and the results of statistical analysis of psycholeptic-associated CD and UC are presented in S2–S7 Tables. In the analysis of CD, significant inverse associations were detected for haloperidol, estazolam, rilmazafone, diazepam, hydroxyzine, and cloxazolam at least three interval (S2–S4 Tables). In the analysis of UC, significant inverse associations were detected for haloperidol, zolpidem, flunitrazepam, zopiclone, diazepam, and hydroxyzine at least three interval (S5–S7 Tables). Consequently, haloperidol, estazolam, rilmazafone, diazepam, hydroxyzine, and cloxazolam were inversely associated with a diagnosis of CD; and haloperidol, zolpidem, flunitrazepam, zopiclone, diazepam, and hydroxyzine were inversely associated with a diagnosis of UC. A summary of the signals detected for psycholeptic-associated CD and UC is presented in S8 Table.

### Disproportionality analysis

We identified 61,383 and 27,375 drug-reaction pairs associated with CD and UC, respectively. The statistical data for psycholeptic-associated CD and UC are presented in S9 and S10 Tables, respectively. In the analysis of CD, significant inverse associations were detected for risperidone, aripiprazole, olanzapine, quetiapine, levomepromazine, haloperidol, chlorpromazine, sulpiride, prochlorperazine, paliperidone, brotizolam, zolpidem, flunitrazepam, triazolam, zopiclone, eszopiclone, phenobarbital, etizolam, diazepam, bromazepam, and hydroxyzine. In the analysis of UC, significant inverse associations were detected for risperidone, aripiprazole, olanzapine, quetiapine, haloperidol, chlorpromazine, promethazine, prochlorperazine, paliperidone, zolpidem, eszopiclone, alprazolam, diazepam, lorazepam, and hydroxyzine. A summary of the FAERS analysis is presented in S11 Table.

### Detection of drug-repositioning signals

A summary of the drug-repositioning signals detected for psycholeptic-associated CD and UC is presented in Table 1. The analyses using three different methodologies indicates that haloperidol, diazepam, and hydroxyzine were inversely associated with CD, and haloperidol, zolpidem, diazepam, and hydroxyzine were inversely associated with UC. These inverse signals were defined as the drug-repositioning signals in the present study. We therefore focused on haloperidol, diazepam, and hydroxyzine to validate drug-repositioning signals using gene expression analysis. The results of statistical analysis of haloperidol, diazepam, and hydroxyzine are presented in Table 2.

**Table 1:** Summary of drug-repositioning signals.

		Crohn's disease			Ulcerative colitis		
		SSA	Disproportionality analysis		SSA	Disproportionality analysis	
		ASR	ROR	IC	ASR	ROR	IC
N05A	Risperidone	-	▼	▼	-	▼	▼
	Aripiprazole	-	▼	▼	-	▼	▼
	Olanzapine	-	▼	▼	-	▼	▼
	Quetiapine	-	▼	▼	-	▼	▼
	Levomepromazine	-	▼	▼	-	-	-
	Haloperidol	▼	▼	▼	▼	▼	▼
	Chlorpromazine	-	▼	▼	-	▼	▼
	Blonaserin	-	-	-	-	-	-
	Perospirone	-	-	-	-	-	-
	Zotepine	-	-	-	-	-	-
	Sulpiride	-	▼	▼	-	-	-
	Prochlorperazine	-	▼	▼	-	▼	▼
	Paliperidone	-	▼	▼	-	▼	▼
	Bromperidol	-	-	-	-	-	-
	Perphenazine	-	-	-	-	-	-
	Propericiazine	-	-	-	-	-	-
	Tiapride	-	-	-	-	-	-
N05B	Ramelteon	-	-	-	-	-	-
	Brotizolam	-	▼	▼	-	-	-
	Zolpidem	-	▼	▼	▼	▼	▼
	Flunitrazepam	-	▼	▼	▼	-	-
	Triazolam	-	▼	▼	-	-	-
	Nitrazepam	-	△	△	-	△	△
	Zopiclone	-	▼	▼	▼	-	-
	Estazolam	▼	-	-	-	-	-
	Rilmazafone	▼	-	-	-	-	-
	Eszopiclone	-	▼	▼	-	▼	▼
	Lormetazepam	-	-	-	-	-	-
	Phenobarbital	-	▼	▼	-	-	-
	Quazepam	-	-	-	-	-	-
	Triclofos	-	-	-	-	△	-
	Suvorexant	-	-	-	△	-	-
	Flurazepam	-	-	-	-	-	-
	Bromovalerylurea	-	-	-	-	-	-
	Nimetazepam	-	-	-	-	-	-
	Amobarbital	-	-	-	-	-	-
	Chloral hydrate	-	-	-	-	-	-
	Haloxazolam	-	-	-	-	-	-
N05C	Etizolam	-	▼	▼	-	-	-
	Alprazolam	-	-	-	-	▼	▼
	Ethyl loflazepate	-	-	-	-	-	-
	Diazepam	▼	▼	▼	▼	▼	▼
	Lorazepam	-	-	-	-	▼	▼
	Clotiazepam	-	-	-	-	-	-
	Bromazepam	-	▼	▼	-	-	-
	Hydroxyzine	▼	▼	▼	▼	▼	▼
	Cloxazolam	▼	-	-	-	-	-
	Dandospirone	-	-	-	-	-	-
	Tofisopam	-	-	-	-	-	-

SSA, Sequence symmetry analysis; ASR, Adjusted sequence ratio; ROR, Reporting odds ratio; IC; Information component; △, risk signal; ▼, drug-repositioning signal

**Table 2:** Drug-repositioning signals validated by gene expression analysis.

	Crohn's disease				Ulcerative colitis			
	Symmetry analysis		Disproportionality analysis		Symmetry analysis		Disproportionality analysis	
	Interval (month)	ASR(95%CI)	ROR (95%CI)	IC (95%CI)	Interval (month)	ASR (95%CI)	ROR (95%CI)	IC (95%CI)
Haloperidol	6	0.18 (0.02–0.80)	0.14 (0.07–0.25)	−2.73 (−3.6 to −1.86)	6	0.44 (0.15–1.12)	0.22 (0.1–0.45)	−2.05 (−3.07 to −1.03)
	12	0.16 (0.02–0.71)			12	0.34 (0.13–0.80)		
	24	0.20 (0.04–0.71)			24	0.30 (0.13–0.66)		
	36	0.18 (0.03–0.64)			36	0.29 (0.13–0.61)		
Diazepam	6	0.39 (0.26–0.58)	0.62 (0.51–0.73)	−0.69 (−0.94 to −0.42)	6	0.57 (0.44–0.73)	0.43 (0.31–0.59)	−1.19 (−1.64 to −0.72)
	12	0.54 (0.39–0.74)			12	0.67 (0.54–0.82)		
	24	0.68 (0.51–0.90)			24	0.69 (0.58–0.83)		
	36	0.66 (0.51–0.86)			36	0.70 (0.59–0.83)		
Hydroxyzine	6	0.52 (0.36–0.75)	0.67 (0.51–0.85)	−0.58 (−0.93 to −0.21)	6	0.57 (0.40–0.80)	0.54 (0.36–0.81)	−0.85 (−1.43 to −0.25)
	12	0.58 (0.42–0.80)			12	0.66 (0.50–0.87)		
	24	0.59 (0.44–0.78)			24	0.69 (0.54–0.88)		
	36	0.61 (0.47–0.81)			36	0.67 (0.54–0.84)		

ASR, Adjusted sequence ratio; ROR, Reporting odds ratio; IC; Information component

Haloperidol, diazepam, and hydroxyzine met the criteria for all indices (ASR, ROR, and IC). Drug-repositioning signals detected for haloperidol, diazepam, and hydroxyzine were validated by gene expression analysis.

#### Validation of drug-repositioning signals using gene expression analysis

We utilized the transcriptome data available from open resources and searched for supportive evidence acquired from genomic data to further validate drug-repositioning signals extracted from mining RWD. First, we searched curated studies available in the NextBio database (BaseSpace Correlation Engine) of gene expression data for haloperidol, diazepam, hydroxyzine, tiapride, CD, and UC. Tiapride was not significantly associated with IBD according to the results of SSA and DPA: 1) there is no risk signal in either SSA or DA analysis. 2) There is also no drug-repositioning signal in either SSA or DA analysis. Therefore, tiapride served as a negative control for gene expression analysis, to ensure that the drug-repositioning signals from RWD mining were true positives (Table 1).

The NextBio database is a commercial tool which converts raw data from open resources to gene signatures associated with a biological condition and includes data quality control and statistical analysis. Using datasets extracted through NextBio, one can compare gene expression data originally acquired from different studies. To minimize background noise and ensure curated gene expression profiles of the high quality assays, we set the criteria as follows: 1) human mRNA expression data; 2) data obtained through comparison of compound-treatment vs vehicle-control group or affected tissues from patients vs normal controls, or both; and 3) high signal-to-noise ratios. Five microarray studies met our inclusion criteria described above (S12 Table). Transcriptome data from these studies were extracted for further analysis to identify positive correlations among haloperidol, diazepam, and hydroxyzine. More important, we aimed to identify negative correlations between psycholeptics and IBD using gene expression profiling to identify gene signatures up-regulated in patients with IBD but down-regulated by psycholeptics.

The transcriptome data for query compounds and diseases were extracted as a bioset from NextBio and then subjected to meta-analysis (Fig 1). First, we compared gene signatures associated with drug treatment and IBD to identify common genes and biogroups (i.e., pathways), which were significantly up-regulated in patients with IBD and down-regulated by psycholeptic treatment. The genes identified by the meta-analysis are listed in S13 Table. The top 10 biogroups identified by meta-analysis are listed in Table 3.

**Table 3:** Shared dysregulated biogroups (pathways) in IBD and psycholeptic treatment.

	Haloperidol (↓)		Diazepam (↓)		Hydroxyzine (↓)		Tiapride (↓)		Tiapride (↑)	
	Pathways	Overall Score	Pathways	Overall Score	Pathways	Overall Score	Pathways	Overall Score	Pathways	Overall Score
IBD (↑)	Genes involved in Cytokine Signaling in Immune system	140.5	Genes involved in Cytokine Signaling in Immune system	151.8	Genes involved in Cytokine Signaling in Immune system	141.8	Beta2 integrin cell surface interactions	51.9	Antigen processing and presentation	91.7
	Leishmania infection	105.2	Genes involved in Interferon Signaling	122.5	Cytokine-cytokine receptor interaction	104.6	Validated transcriptional targets of AP1 family members Fra1 and Fra2	46.2	Genes involved in Interferon alpha/beta signaling	88.3
	Cytokine-cytokine receptor interaction	102.7	Leishmania infection	110.0	Antigen processing and presentation	94.6	Genes involved in Cell surface interactions at the vascular wall	44.8	Graft-versus-host disease	81.0
	Genes involved in Innate Immune System	88.2	Cytokine-cytokine receptor interaction	107.0	Genes involved in Interferon alpha/beta signaling	88.8	Genes involved in Synthesis of DNA	37.8	Natural killer cell mediated cytotoxicity	66.6
	Genes involved in Peptide ligand-binding receptors	62.8	Genes involved in Interferon alpha/beta signaling	88.4	Chemokine signaling pathway	83.4	Genes involved in Metabolism of carbohydrates	37.4	Genes involved in Antigen processing-Cross presentation	66.3
	Complement and coagulation cascades	62.6	Genes involved in Chemokine receptors bind chemokines	79.2	Genes involved in Chemokine receptors bind chemokines	82.3	Genes involved in Cell Cycle, Mitotic	32.6	Genes involved in ER-Phagosome pathway	65.2
	Genes involved in GPCR ligand binding	61.3	Genes involved in Platelet activation, signaling and aggregation	69.1	IL12-mediated signaling events	66.5	Genes involved in Cell Cycle	31.3	IL12-mediated signaling events	65.1
	Genes involved in Response to elevated platelet cytosolic Ca2+	56.9	Genes involved in Peptide ligand-binding receptors	67.9	Genes involved in Peptide ligand-binding receptors	66.0	Genes involved in M/G1 Transition	28.0	Genes involved in Integrin cell surface interactions	59.1
	Genes involved in Class A/1 (Rhodopsin-like receptors)	55.1	Natural killer cell mediated cytotoxicity	66.2	Genes involved in GPCR ligand binding	62.0	Genes involved in DNA Replication	27.7	CXCR4-mediated signaling events	57.8
	Pathways in cancer	51.0	Adhesion and Diapedesis of Granulocytes	63.0	Genes involved in Response to elevated platelet cytosolic Ca2+	57.1	Genes involved in Mitotic G1-G1/S phases	27.4	Leukocyte transendothelial migration	56.0

IBD: For each compound, biosets generated from compound treatment together with biosets from CD and UC patient-derived samples were subjected to meta-analysis to identify biogroups, which were up-regulated in IBD but down-regulated when patients were treated with psycholeptics. The top 10 dysregulated biogroups associated with each compound are listed here. Controls included biogroups, which were up-regulated in IBD, are listed as up-regulated or down-regulated by tiapride. Biogroups that appear in the results for all hit compounds or were common between any hit compound and control compound are highlighted. The overall score is an internal score, calculated using the meta-analysis, serves as a tool indicating a correlation between dysregulation of a biogroup and the analyzed biosets. As described in 'materials and methods', a correlation score is generated to indicate the strength of association between a biogroup/pathway and a disease or compound treatment. The proprietary unique algorithm in meta-analysis to calculate the correlation score is not disclosed to user. More specifically, for each pathway, three correlation scores were generated, which are the correlation scores between a pathway and UC, between a pathway and CD, and between a pathway and haloperidol treatment, respectively. Overall score is the sum-up of three correlation scores above. Higher overall score for a pathway indicates its stronger association with both IBD and haloperidol treatment. To be noted, outcomes from meta-analysis must be read comprehensively. Follow-up literation mining, *in vitro* and *in vivo* studies are required to validate and translate the findings.

Genes encoding cytokines and chemokines were commonly found in gene signatures of the psycholeptic-treatment and disease groups, although not in the tiapride-treated group. The biogroup’s genes involved in cytokine signaling in immune system and cytokine-cytokine receptor interactions were commonly identified in the compound-treatment and disease groups. The association of tiapride with the IBD–associated biogroups was generally weak, according to the overall score of meta-analysis. The results of the meta-analysis of the control compound tiapride had no significant effect on IBD-associated genes and pathways, strongly suggesting that the drug-repositioning signal from RWD mining is more likely true positive. These results are consistent with studies showing that cytokines and chemokines play crucial roles in the pathologies of CD and UC [38, 39] and increased the confidence levels of the findings acquired using the FAERS and JMDC claim databases.

**Fig 2:**
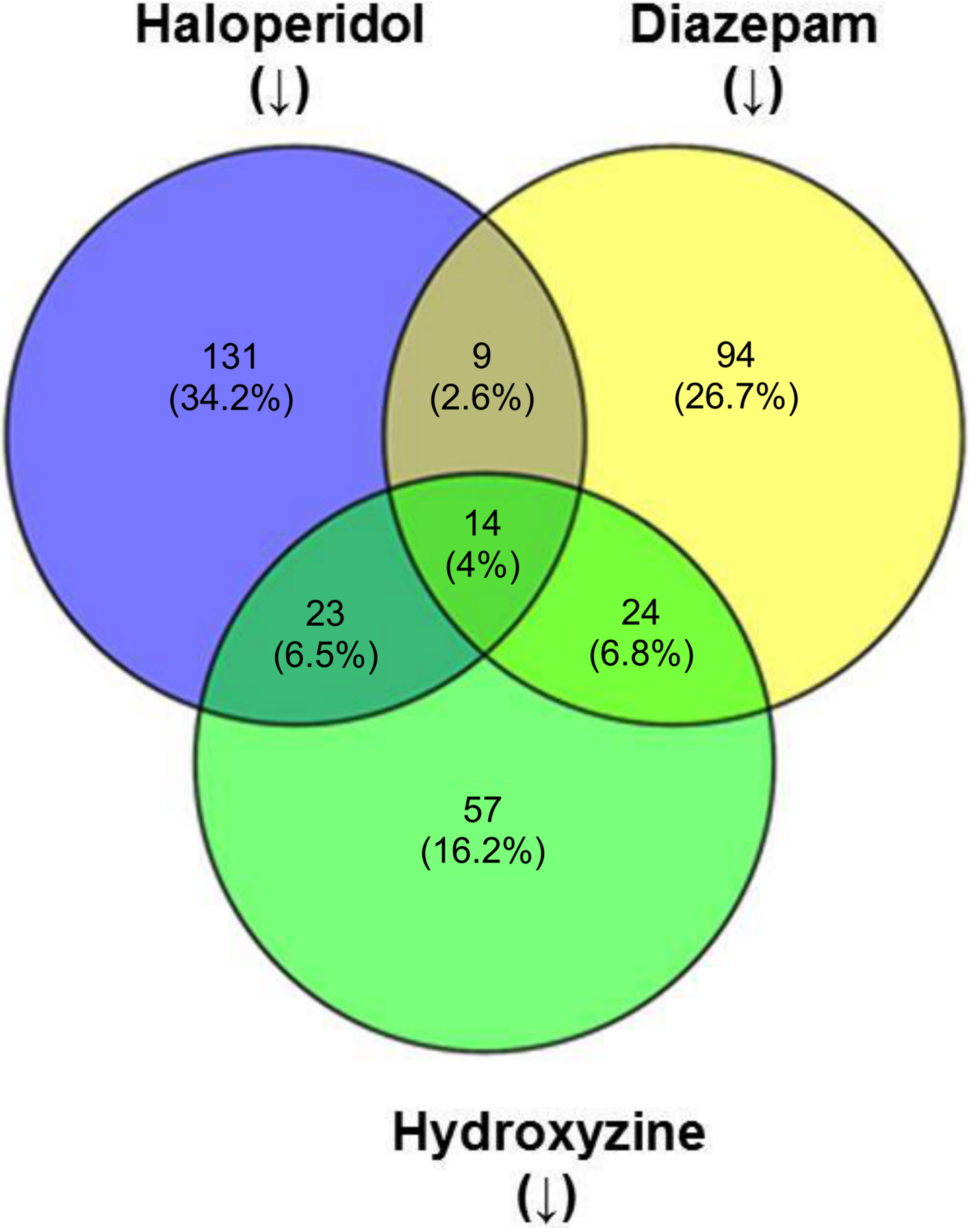
Overlap of related compounds/drugs determined using CMAP analysis. CMAP analysis of gene signatures of a query compound (e.g., haloperidol), reveals compounds with gene signatures that are negatively or positively associated with haloperidol at the cutoff of p <0.05. The Venn diagram shows that selected compounds/drugs are commonly found in the results of CAMP analysis of query compounds.

Connectivity MAP (CMAP, Broad Institute) analyses were conducted using differentially expressed genes. Drugs with gene-signatures that matched those of a query compound were short-listed from the CMAP database [19, 21]. Using the cutoff p <0.05, 177, 141, and 118 drug signatures negatively or positively correlated with signatures for haloperidol, diazepam, and hydroxyzine, respectively (Fig 2). Fourteen compounds were shared among CMAP analyses results for haloperidol, diazepam, and hydroxyzine (Table 4). These compounds are anti-infectious agents, antidepressive/antipsychotic agents, or anti-inflammatory agents, according to the MESH pharmacological classification. Although these compounds differ in their targets and mechanisms of action, their gene signatures used for CMAP analysis share features in common with those of haloperidol, diazepam, and hydroxyzine.

**Table 4:** Overlapping compounds/drugs identified from CMAP analysis.

Compound name	Pharmacological Classification (MeSH)	Indication (Drugbank)
Monensin	Antifungal Agent	N/A
	Antiprotozoal Agents	
	Proton Ionophores	
	Coccidiostats	
	Sodium Ionophores	
15-Delta Prostaglandin J2	Immunologic Factors	N/A
Lasalocid	Ionophores	N/A
	Anti-Bacterial Agents	
	Coccidiostats	
Metixene	N/A	symptomatic treatment of parkinsonism
Thioproperazine	N/A	Schizophrenia
		Manic syndromes
8-Azaguanine	Antimetabolites	N/A
	Antineoplastic	
Maprotiline	Antidepressive Agents, Second-Generation	Anxiety
	Adrenergic Uptake Inhibitors	Depressive illness
		Dysthymic Disorder
		Major Depressive Disorder (MDD)
		Manic depressive illness
Loperamide	Antidiarrheals	Chronic Diarrhea
		Diarrhea
		Intestinal stoma leak
		Traveler's Diarrhea
Benzamil	N/A	N/A
Hexetidine	Anti-Infective Agents, Local	N/A
	Antifungal Agents	
Perphenazine	Antipsychotic Agents;	Schizophrenia
	Dopamine Antagonists	Severe Nausea and vomiting
Amitriptyline	Antidepressive Agents, Tricyclic	Acute Depression
	Adrenergic Uptake Inhibitors	ADHD
	Analgesics, Non-Narcotic	Anorexia Nervosa (AN)
		Bulimia
		Depression
		Diabetic Neuropathies
		Insomnia
		Irritable Bowel Syndrome (IBS)
		Migraines
		Sleep disorders and disturbances
Oxyphenbutazone	Anti-Inflammatory Agents, Non-Steroidal	N/A
Parthenolide	Anti-Inflammatory Agents, Non-Steroidal	N/A

The 14 compounds/drugs overlapped the results of CAMP analysis of gene signatures of haloperidol, diazepam, and hydroxyzine. Compound names, MESH pharmacological classifications, and indications are summarized. Information for indications was acquired from the DrugBank database (https://www.drugbank.ca/).

Interestingly, certain hit compounds identified using CMAP were related to IBD, indicated by direct evidence from clinical and preclinical studies or by indirect evidence for the perturbation of signaling pathways that contribute to the pathogenesis of IBD. For example, loperamide, a common over-the-counter treatment for diarrhea, is used to treat chronic diarrhea associated with IBD [40]. Thioproperazine, a dopamine D2-receptor antagonist and antipsychotic drug, dramatically improves the health of patients with UC, according to multiple findings [41]. Parthenolide, a potent inhibitor of the NF-κB signaling pathway, clinically and histologically ameliorates dextran sulfate sodium (DSS)-induced colitis in mice [42]. Further, parthenolide is the only compound among 14 with a signature associated with the control tiapride. Moreover, the results of CMAP analysis indicate that parthenolide negatively correlated with tiapride, indicating a trend of tiapride to worsen IBD through similar pathways that are down-regulated by haloperidol, diazepam, and hyrdoxyzine (Table 4). Further, 15-deoxy-delta 12,14-prostaglandin J2 inhibits the NF-κB signaling pathway at multiple steps [43].

Cytokine expression and signaling, which are significantly up-regulated in patients with IBD, are commonly down-regulated upon exposure to haloperidol, diazepam, and hydroxyzine. Selected CMAP-hit compounds, which share gene signatures similar to those of haloperidol, diazepam, and hydroxyzine, ameliorate IBD. Moreover, certain CMAP-hit compounds inhibit the NF-κB signaling pathway, which is implicated in the pathogenesis of IBD [44]. In addition, because the NF-κB signaling pathway controls the expression of genes in inflammatory responses, including cytokines and chemokines, these findings are also consistent with the results of meta-analysis of transcriptome data that dysregulated cytokine signaling was commonly found in gene signatures of psycholetic-treatment and disease groups [44].

## Discussion

Computational approaches are widely used to effectively and efficiently identify novel therapeutic opportunities [45–48]. The recent common practice is to connect chemical space with genomic data using CMAP and information on AEs [1, 2, 22]. The major approaches to create value from information about AEs are as follows: 1) repurpose the drug according to AEs or 2) repurpose the drug according to the similarities of the patterns of AEs associated with different drugs [1–3]. Here we introduced a novel third approach to generate repositioning possibilities by extracting inverse signals available as byproducts of the analysis of spontaneous AE reports and claims databases.

Our analyses of the FAERS and JMDC claims databases reveal significant inverse signals for CD and UC associated with the psycholeptics haloperidol, diazepam, and hydroxyzine. Although there is no definite clinical evidence, we acquired consistent findings from independent analyses employing different methodologies, algorithms, and databases, suggesting that haloperidol, diazepam, and hydroxyzine is inversely associated with the risks for CD and UC. Therefore, these psycholeptics may serve as candidates for treating CD and UC.

Similar approaches were recently reported to apply these inverse associations, which were obtained using DPA, for identification of potential novel pharmacological effects of drugs. For example, Zhao et al. found that the FAERS database helps identify drugs that may be repurposed to mitigate serious AEs [49]. Further, Nagashima et al. analyzed the FAERS database and found that co-administration of vitamin D reduces the risk of quetiapine–induced hyperglycemia [7]. These reports support the conclusion that a data-mining approach using the FAERS database is useful for identifying new therapeutic benefits of marketed drugs. We therefore aimed to establish a novel, comprehensive, and multi-methodological, unbiased approach to identify potential new pharmacological effects and generate repositioning possibilities with mechanistic relevance. Meanwhile, similar approach using SSA to identify these inverse signals was not reported.

To solidify the findings from SSA and DPA by simultaneously identifying potential modes of action, we conducted gene expression analysis using transcriptome data from an open source. Our independent analytical approaches indicate that the haloperidol, diazepam, and hydroxyzine may improve the treatment of CD and UC. Further, we provide compelling evidence supporting the conclusion that such effect may be through modulation of NF-κB and cytokine-signaling pathways to resolve chronic inflammation and thus restore epithelial homeostasis in patients with IBD.

To ensure that the outcome of the meta-analysis was positive, we evaluated tiapride because it is not associated with IBD according to SSA and DPA, which served as control. We found that tiapride had little effect on pathways associated with IBD or the above-mentioned hit compounds. These results support the conclusion that the outcome of the meta-analysis was positive. Together, our analysis of RWD using a multi-methodological approach employing SSA and DPA, followed by gene expression analysis, we identified potentially drug-repositioning opportunities for haloperidol, diazepam, and hydroxyzine for treating IBD. Gene expression profiling identified the potential mechanistic relevance of these compounds for pathology, consistent with earlier findings.

Future analyses of animal models of CD and UC will likely further validate the opportunities discovered in the present study. Moreover, to determine if our methodology contributes to robust therapeutic value, candidate compounds require validation in multiple models (e.g., DSS-induced colitis, CD4+ T-cell adoptive transfer colitis, and IL-10 KO) [50]. We note that each method has potential limitations. For example, SSA and DPA using RWD raise the possibility that the reported event was not caused by the drug because of the limitation of quality control of the RWD. Thus, not every adverse event or medication error associated with a drug is reported, and the database may contain missing data and frequent misspellings of drugs. The diagnoses listed in the claims databases are provided by physicians and may not always be validated. Numerous studies address differential gene expression associated with chemical perturbation or disease progression. Careful quality control and stringent criteria are required to efficiently capture correlation signals. In current study, we defined drug-repositioning signal as the inverse association confirmed by two independent methods: SSA and DPA with statistical significance. In addition, we selected tiapride as a control compound for gene expression analysis because it is not significantly associated with IBD according to SSA and DPA outcomes. It is of our future subject to further optimize the criteria for reliable inverse signals and selection of control for analysis. To mitigate these limitations, sequential, multiple methodological approaches comprising SSA, DPA, and transcriptome analysis, which were developed here, rationalize the validity of an inverse signal. It is important therefore to gain a comprehensive understanding of outcomes and to carefully examine the potential conclusions through mining the literature and curating the data.

In summary, we demonstrate here the potential of a novel approach to identify candidate alternative therapeutic opportunities for marketed drugs. For this purpose, we conducted an integrative analysis of the FAERS and JMDC claims databases as well as transcriptome data. To the best of our knowledge, this is the first report to demonstrate the utility of inverse signals obtained from SSA and DPA using claims databases and spontaneous adverse event reports databases to identify drug-repositioning signals and rationalized by transcriptomic analysis of gene expression data. A significant outcome of our unique analytical methodology was the identification of an inverse association of haloperidol, diazepam, and hydroxyzine with CD and UC. It is of our future subject to conduct *in vitro* and *in vivo* validation studies of drug-repositioning and translate the findings to the clinic.

## Supporting information

S1 Table Characteristics of the study population of the sequence symmetry analysis (JMDC claims database). (DOCX)

S2 Table Association between psycholeptics (N05A) and Crohn's disease (JMDC claims database). Inverse associations were detected for haloperidol at least three intervals.
(DOCX)

S3 Table Association between psycholeptics (N05B) and Crohn's disease (JMDC claims database). Inverse associations were detected for estazolam and rilmazafone at least three intervals.
(DOCX)

S4 Table Association between psycholeptics (N05C) and Crohn's disease (JMDC claims database). Inverse associations were detected for diazepam, hydroxyzine, and cloxazolam at least three intervals.
(DOCX)

S5 Table Association between psycholeptics (N05A) and ulcerative colitis (JMDC claims database). Inverse associations were detected for haloperidol at least three intervals.
(DOCX)

S6 Table Association between psycholeptics (N05B) and ulcerative colitis (JMDC claims database). Inverse associations were detected for zolpidem, flunitrazepam, zopiclone at least three intervals.
(DOCX)

S7 Table Association between psycholeptics (N05C) and ulcerative colitis (JMDC claims database). Inverse associations were detected for diazepam and hydroxyzine at least three intervals.
(DOCX)

S8 Table Summary of event sequence-symmetry analyses (JMDC claims database). Haloperidol, estazolam, rilmazafone, diazepam, hydroxyzine, and cloxazolam were inversely associated with a diagnosis of CD; and haloperidol, zolpidem, flunitrazepam, zopiclone, diazepam, and hydroxyzine were inversely associated with a diagnosis of UC.
(DOCX)

S9 Table Association between psycholeptics and Crohn's disease (FAERS database). Risperidone, aripiprazole, olanzapine, quetiapine, levomepromazine, haloperidol, chlorpromazine, sulpiride, prochlorperazine, paliperidone, brotizolam, zolpidem, flunitrazepam, triazolam, zopiclone, eszopiclone, phenobarbital, etizolam, diazepam, bromazepam, and hydroxyzine were inversely associated with diagnosis of CD.
(DOCX)

S10 Table Association between psycholeptics and ulcerative colitis (FAERS database). Risperidone, aripiprazole, olanzapine, quetiapine, haloperidol, chlorpromazine, promethazine, prochlorperazine, paliperidone, zolpidem, eszopiclone, alprazolam, diazepam, lorazepam, and hydroxyzine were inversely associated with UC.
(DOCX)

S11 Table Summary of the detection of inverse signals of psycholeptic-associated Crohn's disease and ulcerative colitis (FAERS database). Risperidone, aripiprazole, olanzapine, quetiapine, levomepromazine, haloperidol, chlorpromazine, sulpiride, prochlorperazine, paliperidone, brotizolam, zolpidem, flunitrazepam, triazolam, zopiclone, eszopiclone, phenobarbital, etizolam, diazepam, bromazepam, and hydroxyzine were inversely associated with CD; and risperidone, aripiprazole, olanzapine, quetiapine, haloperidol, chlorpromazine, promethazine, prochlorperazine, paliperidone, zolpidem, eszopiclone, alprazolam, diazepam, lorazepam, and hydroxyzine were inversely associated with UC.
(DOCX)

S12 Table Microarray datasets for IBD and compound treatment. Gene expression microarray data were extracted using the NextBio database for bioinformatics analysis. The NextBio database integrates raw data from the open resource GEO by a normalized ranking approach and stores processed data as datasets with a NextBio internal ID. Datasets extracted using the NextBio database are applicable for comparisons of data from different studies. The inclusion criteria for datasets in this study were as follows: 1) mRNA expression data of humans; 2) comparison of compound treatment vs a vehicle control or affected tissue from patients vs a normal control; 3) high signal-to-noise ratio. Detailed information of experimental settings for data acquisition is described.
(DOCX)

S13 Table Differentially expressed genes (DEGs) shared between IBD and treatment with psycholeptics. For each compound, the bioset generated from compound treatment together with biosets from samples acquired from patients with CD or UC were subjected to meta-analysis to identify for DEGs, which were up-regulated in IBD but down-regulated by psycholeptic treatment. DEGs, which were up-regulated in IBD listed as either up-regulated or down-regulated by tiapride, served as controls. The overall score is an internal score, calculated using the meta-analysis tool, indicates a correlation between DEGs and the analyzed biosets. DEGs with p <0.05 are listed.
(DOCX)
