# Supplementary material for: An integrative approach using real-world data to identify alternative therapeutic uses of existing drugs
Source: PLoS One. 2018 Oct 9;13(10):e0204648. doi: 10.1371/journal.pone.0204648 (PMC6177143; doi:10.1371/journal.pone.0204648)
Supplement: S1 Table — (DOCX) [file pone.0204648.s001.docx]

S1 Table. Characteristics of the study population of the sequence symmetry analysis (JMDC claims database)

| Drugs | Users | Claims including antipsychotic drugs | Incident users | Age (mean ± SD) |
| --- | --- | --- | --- | --- |
| N05A |  |  |  |  |
| Risperidone | 18,709 | 405,388 | 10,819 | 34.2±17.9 |
| Aripiprazole | 20,617 | 395,455 | 14,186 | 35.0±13.9 |
| Olanzapine | 15,582 | 354,578 | 9,905 | 38.0±13.5 |
| Quetiapine | 11,077 | 294,531 | 6,880 | 38.7±14.1 |
| Levomepromazine | 6,864 | 179,942 | 3,889 | 37.6±13.4 |
| Haloperidol | 9,907 | 138,514 | 6,797 | 46.6±16.3 |
| Chlorpromazine | 6,319 | 108,588 | 4,000 | 37.9±17.5 |
| Blonaserin | 3,239 | 75,288 | 2,103 | 36.0±13.5 |
| Perospirone | 3,100 | 60,252 | 1,900 | 36.4±13.6 |
| Zotepine | 1,170 | 45,894 | 612 | 38.0±12.9 |
| Sulpiride | 3,470 | 37,342 | 2,251 | 39.1±12.5 |
| Prochlorperazine | 8,500 | 36,848 | 7,197 | 47.2±14.9 |
| Paliperidone | 1,364 | 34,025 | 910 | 35.0±14.0 |
| Bromperidol | 783 | 27,281 | 299 | 36.8±12.8 |
| Perphenazine | 1,847 | 23,009 | 1,162 | 36.9±12.9 |
| Propericiazine | 710 | 17,850 | 348 | 26.0±17.1 |
| Tiapride | 799 | 12,869 | 533 | 56.1±15.5 |
| N05B |  |  |  |  |
| Ramelteon | 13,234 | 116,307 | 10,690 | 39.1±17.0 |
| Brotizolam | 82,119 | 1,017,103 | 54,573 | 44.3±13.9 |
| Zolpidem | 96,553 | 947,353 | 66,544 | 45.0±13.9 |
| Flunitrazepam | 65,902 | 810,357 | 45,783 | 46.0±12.6 |
| Triazolam | 30,571 | 462,013 | 17,893 | 43.5±13.8 |
| Nitrazepam | 14,857 | 266,553 | 8,932 | 41.2±14.2 |
| Zopiclone | 21,304 | 242,366 | 14,366 | 45.2±14.0 |
| Estazolam | 13,395 | 145,844 | 8,499 | 43.2±13.0 |
| Rilmazafone | 15,987 | 117,380 | 11,849 | 45.3±13.5 |
| Eszopiclone | 13,986 | 108,753 | 11,852 | 43.8±13.6 |
| Lormetazepam | 7,443 | 93,783 | 4,938 | 41.2±12.8 |
| Phenobarbital | 8,318 | 90,462 | 4,871 | 22.0±20.9 |
| Quazepam | 5,775 | 85,727 | 3,769 | 40.3±12.0 |
| Triclofos | 23,053 | 61,505 | 13,847 | 4.1±5.0 |
| Suvorexant | 6,076 | 28,169 | 5,785 | 44.8±13.3 |
| Flurazepam | 1,002 | 21,077 | 628 | 41.0±11.2 |
| Bromovalerylurea | 5,435 | 19,699 | 4,169 | 32.8±17.6 |
| Nimetazepam | 1,175 | 16,966 | 777 | 38.5±12.1 |
| Amobarbital | 602 | 14,491 | 360 | 39.6±11.8 |
| Chloral hydrate | 6,748 | 13,875 | 5,321 | 4.4±6.1 |
| Haloxazolam | 597 | 10,217 | 407 | 40.3±10.4 |
| N05C |  |  |  |  |
| Etizolam | 119,988 | 1,356,384 | 76,230 | 43.4±13.3 |
| Alprazolam | 53,934 | 686,882 | 33,417 | 40.3±12.8 |
| Ethyl loflazepate | 43,901 | 534,115 | 29,299 | 40.5±12.8 |
| Diazepam | 108,453 | 398,779 | 84,620 | 43.6±15.3 |
| Lorazepam | 30,673 | 384,793 | 20,320 | 39.1±12.7 |
| Clotiazepam | 45,296 | 361,878 | 32,494 | 40.9±13.7 |
| Bromazepam | 20,980 | 323,259 | 12,281 | 37.3±13.4 |
| Hydroxyzine | 80,657 | 164,226 | 66,670 | 39.8±17.6 |
| Cloxazolam | 8,164 | 113,659 | 4,937 | 39.2±12.5 |
| Dandospirone | 9,604 | 77,942 | 7,114 | 39.1±13.9 |
| Tofisopam | 13,407 | 75,084 | 10,014 | 41.3±14.0 |

Incident users: Number of patients who received their first prescription for drug

SD: Standard deviation
