# Supplementary material for: An integrative approach using real-world data to identify alternative therapeutic uses of existing drugs
Source: PLoS One. 2018 Oct 9;13(10):e0204648. doi: 10.1371/journal.pone.0204648 (PMC6177143; doi:10.1371/journal.pone.0204648)
Supplement: S2 Table — Inverse associations were detected for haloperidol at least three intervals. (DOCX) [file pone.0204648.s002.docx]

S2 Table. Association between psycholeptics (N05A) and Crohn's disease (JMDC claims database)

Inverse associations were detected for haloperidol at least three intervals.

| Drugs | Incident users | Cocomitant users | Simultaneous start | interval (months) | last | first | Crude SR | Null-Effect SR | Adjusted SR | 95%CI | |
| --- | --- | --- | --- | --- | --- | --- | --- | --- | --- | --- | --- |
|  |  |  |  |  |  |  |  |  |  | Lower | Upper |
| Risperidone | 10,819 | 35 | 2 | 6 | 2 | 6 | 0.33 | 1.02 | 0.33 | 0.03 | 1.82 |
|  |  |  |  | 12 | 4 | 10 | 0.40 | 1.04 | 0.38 | 0.09 | 1.33 |
|  |  |  |  | 24 | 7 | 12 | 0.58 | 1.07 | 0.54 | 0.18 | 1.50 |
|  |  |  |  | 36 | 10 | 13 | 0.77 | 1.10 | 0.70 | 0.28 | 1.73 |
| Aripiprazole | 14,186 | 36 | 0 | 6 | 5 | 6 | 0.83 | 1.02 | 0.82 | 0.20 | 3.21 |
|  |  |  |  | 12 | 9 | 7 | 1.29 | 1.03 | 1.25 | 0.42 | 3.96 |
|  |  |  |  | 24 | 14 | 11 | 1.27 | 1.02 | 1.25 | 0.53 | 3.04 |
|  |  |  |  | 36 | 14 | 12 | 1.17 | 0.98 | 1.19 | 0.51 | 2.80 |
| Olanzapine | 9,905 | 38 | 4 | 6 | 5 | 2 | 2.50 | 1.04 | 2.40 | 0.39 | 25.24 |
|  |  |  |  | 12 | 8 | 5 | 1.60 | 1.07 | 1.49 | 0.43 | 5.79 |
|  |  |  |  | 24 | 10 | 10 | 1.00 | 1.13 | 0.89 | 0.33 | 2.37 |
|  |  |  |  | 36 | 12 | 13 | 0.92 | 1.16 | 0.80 | 0.33 | 1.89 |
| Quetiapine | 6,880 | 32 | 0 | 6 | 4 | 2 | 2.00 | 1.04 | 1.93 | 0.28 | 21.36 |
|  |  |  |  | 12 | 8 | 8 | 1.00 | 1.06 | 0.95 | 0.31 | 2.89 |
|  |  |  |  | 24 | 11 | 11 | 1.00 | 1.09 | 0.92 | 0.36 | 2.33 |
|  |  |  |  | 36 | 14 | 12 | 1.17 | 1.12 | 1.04 | 0.45 | 2.47 |
| Levomepromazine | 3,889 | 15 | 0 | 6 | 2 | 1 | 2.00 | 1.05 | 1.91 | 0.10 | 112.57 |
|  |  |  |  | 12 | 3 | 1 | 3.00 | 1.08 | 2.77 | 0.22 | 145.56 |
|  |  |  |  | 24 | 4 | 6 | 0.67 | 1.13 | 0.59 | 0.12 | 2.48 |
|  |  |  |  | 36 | 6 | 6 | 1.00 | 1.16 | 0.86 | 0.23 | 3.23 |
| Haloperidol | 6,797 | 28 | 5 | 6 | 2 | 11 | 0.18 | 1.04 | 0.18 | 0.02 | 0.80 |
|  |  |  |  | 12 | 2 | 12 | 0.17 | 1.05 | 0.16 | 0.02 | 0.71 |
|  |  |  |  | 24 | 3 | 14 | 0.21 | 1.08 | 0.20 | 0.04 | 0.71 |
|  |  |  |  | 36 | 3 | 15 | 0.20 | 1.10 | 0.18 | 0.03 | 0.64 |
| Chlorpromazine | 4,000 | 9 | 0 | 6 | 1 | 3 | 0.33 | 1.02 | 0.33 | 0.01 | 4.07 |
|  |  |  |  | 12 | 1 | 5 | 0.20 | 1.04 | 0.19 | 0.00 | 1.72 |
|  |  |  |  | 24 | 1 | 6 | 0.17 | 1.08 | 0.15 | 0.00 | 1.27 |
|  |  |  |  | 36 | 1 | 6 | 0.17 | 1.12 | 0.15 | 0.00 | 1.23 |
| Blonaserin | 2,103 | 4 | 0 | 6 | 1 | 2 | 0.50 | 1.04 | 0.48 | 0.01 | 9.24 |
|  |  |  |  | 12 | 1 | 3 | 0.33 | 1.06 | 0.31 | 0.01 | 3.90 |
|  |  |  |  | 24 | 1 | 3 | 0.33 | 1.11 | 0.30 | 0.01 | 3.74 |
|  |  |  |  | 36 | 1 | 3 | 0.33 | 1.14 | 0.29 | 0.01 | 3.64 |
| Perospirone | 1,900 | 10 | 0 | 6 | 1 | 0 | - | 1.03 | - | ‐ | ‐ |
|  |  |  |  | 12 | 1 | 4 | 0.25 | 1.06 | 0.24 | 0.00 | 2.39 |
|  |  |  |  | 24 | 2 | 5 | 0.40 | 1.11 | 0.36 | 0.03 | 2.20 |
|  |  |  |  | 36 | 2 | 5 | 0.40 | 1.14 | 0.35 | 0.03 | 2.13 |
| Zotepine | 612 | 1 | 0 | 6 | 0 | 0 | - | 1.07 | - | ‐ | ‐ |
|  |  |  |  | 12 | 0 | 0 | - | 1.10 | - | ‐ | ‐ |
|  |  |  |  | 24 | 1 | 0 | - | 1.18 | - | ‐ | ‐ |
|  |  |  |  | 36 | 1 | 0 | - | 1.24 | - | ‐ | ‐ |
| Sulpiride | 2,251 | 17 | 2 | 6 | 2 | 2 | 1.00 | 1.02 | 0.98 | 0.07 | 13.47 |
|  |  |  |  | 12 | 3 | 3 | 1.00 | 1.04 | 0.96 | 0.13 | 7.16 |
|  |  |  |  | 24 | 3 | 4 | 0.75 | 1.08 | 0.69 | 0.10 | 4.09 |
|  |  |  |  | 36 | 4 | 5 | 0.80 | 1.12 | 0.71 | 0.14 | 3.31 |
| Prochlorperazine | 7,197 | 17 | 1 | 6 | 1 | 1 | 1.00 | 1.01 | 0.99 | 0.01 | 78.04 |
|  |  |  |  | 12 | 2 | 1 | 2.00 | 1.01 | 1.98 | 0.10 | 116.66 |
|  |  |  |  | 24 | 4 | 3 | 1.33 | 1.01 | 1.32 | 0.22 | 8.98 |
|  |  |  |  | 36 | 5 | 4 | 1.25 | 1.00 | 1.24 | 0.27 | 6.27 |
| Paliperidone | 910 | 2 | 0 | 6 | 0 | 0 | - | 1.09 | - | ‐ | ‐ |
|  |  |  |  | 12 | 1 | 1 | 1.00 | 1.15 | 0.87 | 0.01 | 68.02 |
|  |  |  |  | 24 | 1 | 1 | 1.00 | 1.29 | 0.77 | 0.01 | 60.67 |
|  |  |  |  | 36 | 1 | 1 | 1.00 | 1.39 | 0.72 | 0.01 | 56.53 |
| Bromperidol | 299 | 0 | 0 | 6 | 0 | 0 | - | 1.11 | - | ‐ | ‐ |
|  |  |  |  | 12 | 0 | 0 | - | 1.21 | - | ‐ | ‐ |
|  |  |  |  | 24 | 0 | 0 | - | 1.37 | - | ‐ | ‐ |
|  |  |  |  | 36 | 0 | 0 | - | 1.42 | - | ‐ | ‐ |
| Perphenazine | 1,162 | 5 | 1 | 6 | 1 | 0 | - | 1.03 | - | ‐ | ‐ |
|  |  |  |  | 12 | 2 | 0 | - | 1.05 | - | ‐ | ‐ |
|  |  |  |  | 24 | 3 | 1 | 3.00 | 1.11 | 2.71 | 0.22 | 142.10 |
|  |  |  |  | 36 | 3 | 1 | 3.00 | 1.16 | 2.59 | 0.21 | 136.01 |
| Propericiazine | 348 | 0 | 0 | 6 | 0 | 0 | - | 1.06 | - | ‐ | ‐ |
|  |  |  |  | 12 | 0 | 0 | - | 1.09 | - | ‐ | ‐ |
|  |  |  |  | 24 | 0 | 0 | - | 1.17 | - | ‐ | ‐ |
|  |  |  |  | 36 | 0 | 0 | - | 1.22 | - | ‐ | ‐ |
| Tiapride | 533 | 0 | 0 | 6 | 0 | 0 | - | 1.00 | - | ‐ | ‐ |
|  |  |  |  | 12 | 0 | 0 | - | 1.02 | - | ‐ | ‐ |
|  |  |  |  | 24 | 0 | 0 | - | 1.04 | - | ‐ | ‐ |
|  |  |  |  | 36 | 0 | 0 | - | 1.09 | - | ‐ | ‐ |
