# Supplementary material for: An integrative approach using real-world data to identify alternative therapeutic uses of existing drugs
Source: PLoS One. 2018 Oct 9;13(10):e0204648. doi: 10.1371/journal.pone.0204648 (PMC6177143; doi:10.1371/journal.pone.0204648)
Supplement: S3 Table — Inverse associations were detected for estazolam and rilmazafone at least three intervals. (DOCX) [file pone.0204648.s003.docx]

S3 Table. Association between psycholeptics (N05B) and Crohn's disease (JMDC claims database)

Inverse associations were detected for estazolam and rilmazafone at least three intervals.

|  | Incident users | Cocomitant users | Simultaneous start | interval (months) | last | first | Crude SR | Null-Effect SR | Adjusted SR | 95%CI | |
| --- | --- | --- | --- | --- | --- | --- | --- | --- | --- | --- | --- |
|  |  |  |  |  |  |  |  |  |  | Lower | Upper |
| Ramelteon | 10,690 | 27 | 0 | 6 | 3 | 3 | 1.00 | 1.02 | 0.98 | 0.13 | 7.32 |
|  |  |  |  | 12 | 5 | 6 | 0.83 | 1.03 | 0.81 | 0.20 | 3.20 |
|  |  |  |  | 24 | 6 | 8 | 0.75 | 1.03 | 0.73 | 0.21 | 2.39 |
|  |  |  |  | 36 | 8 | 10 | 0.80 | 1.03 | 0.78 | 0.27 | 2.19 |
| Brotizolam | 54,573 | 176 | 22 | 6 | 20 | 28 | 0.71 | 1.05 | 0.68 | 0.36 | 1.26 |
|  |  |  |  | 12 | 31 | 34 | 0.91 | 1.08 | 0.85 | 0.50 | 1.42 |
|  |  |  |  | 24 | 49 | 50 | 0.98 | 1.13 | 0.87 | 0.58 | 1.32 |
|  |  |  |  | 36 | 58 | 60 | 0.97 | 1.16 | 0.83 | 0.57 | 1.22 |
| Zolpidem | 66,544 | 195 | 17 | 6 | 24 | 28 | 0.86 | 1.04 | 0.82 | 0.46 | 1.47 |
|  |  |  |  | 12 | 36 | 36 | 1.00 | 1.07 | 0.94 | 0.57 | 1.53 |
|  |  |  |  | 24 | 55 | 60 | 0.92 | 1.11 | 0.82 | 0.56 | 1.21 |
|  |  |  |  | 36 | 66 | 72 | 0.92 | 1.14 | 0.80 | 0.57 | 1.14 |
| Flunitrazepam | 45,783 | 325 | 122 | 6 | 34 | 51 | 0.67 | 1.04 | 0.64 | 0.40 | 1.01 |
|  |  |  |  | 12 | 55 | 62 | 0.89 | 1.06 | 0.84 | 0.57 | 1.22 |
|  |  |  |  | 24 | 78 | 73 | 1.07 | 1.10 | 0.97 | 0.70 | 1.35 |
|  |  |  |  | 36 | 91 | 85 | 1.07 | 1.12 | 0.95 | 0.70 | 1.30 |
| Triazolam | 17,893 | 63 | 6 | 6 | 4 | 7 | 0.57 | 1.06 | 0.54 | 0.12 | 2.12 |
|  |  |  |  | 12 | 10 | 13 | 0.77 | 1.10 | 0.70 | 0.27 | 1.72 |
|  |  |  |  | 24 | 15 | 18 | 0.83 | 1.19 | 0.70 | 0.33 | 1.47 |
|  |  |  |  | 36 | 19 | 20 | 0.95 | 1.26 | 0.75 | 0.38 | 1.49 |
| Nitrazepam | 8,932 | 27 | 0 | 6 | 2 | 4 | 0.50 | 1.05 | 0.48 | 0.04 | 3.34 |
|  |  |  |  | 12 | 3 | 7 | 0.43 | 1.08 | 0.40 | 0.07 | 1.73 |
|  |  |  |  | 24 | 9 | 10 | 0.90 | 1.16 | 0.78 | 0.28 | 2.13 |
|  |  |  |  | 36 | 10 | 12 | 0.83 | 1.22 | 0.68 | 0.27 | 1.73 |
| Zopiclone | 14,366 | 53 | 11 | 6 | 5 | 7 | 0.71 | 1.06 | 0.67 | 0.17 | 2.46 |
|  |  |  |  | 12 | 5 | 9 | 0.56 | 1.11 | 0.50 | 0.13 | 1.67 |
|  |  |  |  | 24 | 8 | 13 | 0.62 | 1.20 | 0.51 | 0.19 | 1.34 |
|  |  |  |  | 36 | 11 | 16 | 0.69 | 1.27 | 0.54 | 0.23 | 1.24 |
| Estazolam | 8,499 | 216 | 152 | 6 | 11 | 28 | 0.39 | 1.05 | 0.37 | 0.17 | 0.77 |
|  |  |  |  | 12 | 13 | 31 | 0.42 | 1.09 | 0.38 | 0.18 | 0.75 |
|  |  |  |  | 24 | 19 | 34 | 0.56 | 1.18 | 0.47 | 0.26 | 0.85 |
|  |  |  |  | 36 | 22 | 38 | 0.58 | 1.26 | 0.46 | 0.26 | 0.80 |
| Rilmazafone | 11,849 | 50 | 1 | 6 | 5 | 23 | 0.22 | 1.05 | 0.21 | 0.06 | 0.56 |
|  |  |  |  | 12 | 5 | 24 | 0.21 | 1.09 | 0.19 | 0.06 | 0.51 |
|  |  |  |  | 24 | 7 | 26 | 0.27 | 1.17 | 0.23 | 0.09 | 0.55 |
|  |  |  |  | 36 | 9 | 27 | 0.33 | 1.23 | 0.27 | 0.11 | 0.59 |
| Eszopiclone | 11,852 | 41 | 7 | 6 | 3 | 7 | 0.43 | 0.99 | 0.43 | 0.07 | 1.90 |
|  |  |  |  | 12 | 5 | 9 | 0.56 | 0.95 | 0.58 | 0.15 | 1.94 |
|  |  |  |  | 24 | 5 | 15 | 0.33 | 0.87 | 0.38 | 0.11 | 1.11 |
|  |  |  |  | 36 | 8 | 17 | 0.47 | 0.78 | 0.61 | 0.23 | 1.48 |
| Lormetazepam | 4,938 | 18 | 1 | 6 | 2 | 0 | - | 1.06 | - | ‐ | ‐ |
|  |  |  |  | 12 | 2 | 1 | 2.00 | 1.09 | 1.83 | 0.10 | 107.95 |
|  |  |  |  | 24 | 6 | 3 | 2.00 | 1.16 | 1.73 | 0.37 | 10.70 |
|  |  |  |  | 36 | 7 | 6 | 1.17 | 1.21 | 0.97 | 0.28 | 3.49 |
| Phenobarbital | 4,871 | 5 | 1 | 6 | 1 | 1 | 1.00 | 1.08 | 0.93 | 0.01 | 72.65 |
|  |  |  |  | 12 | 1 | 1 | 1.00 | 1.15 | 0.87 | 0.01 | 68.42 |
|  |  |  |  | 24 | 1 | 1 | 1.00 | 1.26 | 0.80 | 0.01 | 62.43 |
|  |  |  |  | 36 | 1 | 1 | 1.00 | 1.34 | 0.75 | 0.01 | 58.77 |
| Quazepam | 3,769 | 20 | 2 | 6 | 1 | 4 | 0.25 | 1.09 | 0.23 | 0.01 | 2.31 |
|  |  |  |  | 12 | 2 | 4 | 0.50 | 1.15 | 0.43 | 0.04 | 3.02 |
|  |  |  |  | 24 | 3 | 5 | 0.60 | 1.27 | 0.47 | 0.07 | 2.43 |
|  |  |  |  | 36 | 6 | 7 | 0.86 | 1.36 | 0.63 | 0.18 | 2.20 |
| Triclofos | 13,847 | 4 | 0 | 6 | 0 | 1 | 0.00 | 1.05 | 0.00 | ‐ | ‐ |
|  |  |  |  | 12 | 0 | 2 | 0.00 | 1.08 | 0.00 | ‐ | ‐ |
|  |  |  |  | 24 | 1 | 2 | 0.50 | 1.13 | 0.44 | 0.01 | 8.48 |
|  |  |  |  | 36 | 1 | 2 | 0.50 | 1.17 | 0.43 | 0.01 | 8.19 |
| Suvorexant | 5,785 | 9 | 0 | 6 | 1 | 1 | 1.00 | 0.73 | 1.38 | 0.02 | 108.00 |
|  |  |  |  | 12 | 1 | 2 | 0.50 | 0.55 | 0.90 | 0.02 | 17.32 |
|  |  |  |  | 24 | 1 | 4 | 0.25 | 0.32 | 0.77 | 0.02 | 7.81 |
|  |  |  |  | 36 | 1 | 5 | 0.20 | 0.25 | 0.79 | 0.02 | 7.03 |
| Flurazepam | 628 | 1 | 0 | 6 | 0 | 0 | - | 1.08 | - | ‐ | ‐ |
|  |  |  |  | 12 | 0 | 0 | - | 1.16 | - | ‐ | ‐ |
|  |  |  |  | 24 | 0 | 0 | - | 1.29 | - | ‐ | ‐ |
|  |  |  |  | 36 | 0 | 0 | - | 1.39 | - | ‐ | ‐ |
| Bromovalerylurea | 4,169 | 16 | 1 | 6 | 0 | 2 | 0.00 | 1.03 | 0.00 | ‐ | ‐ |
|  |  |  |  | 12 | 1 | 3 | 0.33 | 1.09 | 0.31 | 0.01 | 3.82 |
|  |  |  |  | 24 | 3 | 5 | 0.60 | 1.19 | 0.51 | 0.08 | 2.60 |
|  |  |  |  | 36 | 4 | 6 | 0.67 | 1.28 | 0.52 | 0.11 | 2.20 |
| Nimetazepam | 777 | 4 | 0 | 6 | 0 | 0 | - | 1.14 | - | ‐ | ‐ |
|  |  |  |  | 12 | 0 | 0 | - | 1.22 | - | ‐ | ‐ |
|  |  |  |  | 24 | 2 | 1 | 2.00 | 1.36 | 1.47 | 0.08 | 86.53 |
|  |  |  |  | 36 | 2 | 2 | 1.00 | 1.45 | 0.69 | 0.05 | 9.52 |
| Amobarbital | 360 | 1 | 0 | 6 | 0 | 0 | - | 1.07 | - | ‐ | ‐ |
|  |  |  |  | 12 | 0 | 0 | - | 1.13 | - | ‐ | ‐ |
|  |  |  |  | 24 | 0 | 0 | - | 1.25 | - | ‐ | ‐ |
|  |  |  |  | 36 | 0 | 0 | - | 1.37 | - | ‐ | ‐ |
| Chloral hydrate | 5,321 | 0 | 0 | 6 | 0 | 0 | - | 1.05 | - | ‐ | ‐ |
|  |  |  |  | 12 | 0 | 0 | - | 1.09 | - | ‐ | ‐ |
|  |  |  |  | 24 | 0 | 0 | - | 1.17 | - | ‐ | ‐ |
|  |  |  |  | 36 | 0 | 0 | - | 1.23 | - | ‐ | ‐ |
| Haloxazolam | 407 | 6 | 0 | 6 | 2 | 0 | - | 1.07 | - | ‐ | ‐ |
|  |  |  |  | 12 | 2 | 0 | - | 1.11 | - | ‐ | ‐ |
|  |  |  |  | 24 | 3 | 0 | - | 1.20 | - | ‐ | ‐ |
|  |  |  |  | 36 | 3 | 1 | 3.00 | 1.32 | 2.28 | 0.18 | 119.52 |
