# Supplementary material for: An integrative approach using real-world data to identify alternative therapeutic uses of existing drugs
Source: PLoS One. 2018 Oct 9;13(10):e0204648. doi: 10.1371/journal.pone.0204648 (PMC6177143; doi:10.1371/journal.pone.0204648)
Supplement: S4 Table — Inverse associations were detected for diazepam, hydroxyzine, and cloxazolam at least three intervals. (DOCX) [file pone.0204648.s004.docx]

S4 Table. Association between psycholeptics (N05C) and Crohn's disease (JMDC claims database)

Inverse associations were detected for diazepam, hydroxyzine, and cloxazolam at least three intervals.

| Drugs | Incident users | Cocomitant users | Simultaneous start | interval (months) | last | first | Crude SR | Null-Effect SR | Adjusted SR | 95%CI | |
| --- | --- | --- | --- | --- | --- | --- | --- | --- | --- | --- | --- |
|  |  |  |  |  |  |  |  |  |  | Lower | Upper |
| Etizolam | 76,230 | 192 | 5 | 6 | 17 | 19 | 0.89 | 1.05 | 0.85 | 0.42 | 1.73 |
|  |  |  |  | 12 | 31 | 34 | 0.91 | 1.09 | 0.84 | 0.50 | 1.41 |
|  |  |  |  | 24 | 53 | 52 | 1.02 | 1.15 | 0.89 | 0.59 | 1.33 |
|  |  |  |  | 36 | 67 | 61 | 1.10 | 1.20 | 0.92 | 0.64 | 1.32 |
| Alprazolam | 33,417 | 97 | 6 | 6 | 11 | 11 | 1.00 | 1.05 | 0.95 | 0.38 | 2.43 |
|  |  |  |  | 12 | 18 | 16 | 1.13 | 1.08 | 1.04 | 0.50 | 2.18 |
|  |  |  |  | 24 | 29 | 23 | 1.26 | 1.13 | 1.11 | 0.62 | 2.01 |
|  |  |  |  | 36 | 31 | 32 | 0.97 | 1.18 | 0.82 | 0.49 | 1.39 |
| Ethyl loflazepate | 29,299 | 102 | 5 | 6 | 14 | 11 | 1.27 | 1.04 | 1.22 | 0.52 | 2.97 |
|  |  |  |  | 12 | 19 | 21 | 0.90 | 1.07 | 0.84 | 0.43 | 1.65 |
|  |  |  |  | 24 | 27 | 33 | 0.82 | 1.13 | 0.72 | 0.42 | 1.24 |
|  |  |  |  | 36 | 32 | 44 | 0.73 | 1.18 | 0.62 | 0.38 | 0.99 |
| Diazepam | 84,620 | 574 | 291 | 6 | 37 | 91 | 0.41 | 1.04 | 0.39 | 0.26 | 0.58 |
|  |  |  |  | 12 | 61 | 106 | 0.58 | 1.07 | 0.54 | 0.39 | 0.74 |
|  |  |  |  | 24 | 90 | 119 | 0.76 | 1.12 | 0.68 | 0.51 | 0.90 |
|  |  |  |  | 36 | 101 | 133 | 0.76 | 1.15 | 0.66 | 0.51 | 0.86 |
| Lorazepam | 20,320 | 54 | 0 | 6 | 11 | 5 | 2.20 | 1.04 | 2.12 | 0.68 | 7.78 |
|  |  |  |  | 12 | 13 | 9 | 1.44 | 1.06 | 1.37 | 0.54 | 3.62 |
|  |  |  |  | 24 | 20 | 15 | 1.33 | 1.09 | 1.22 | 0.60 | 2.57 |
|  |  |  |  | 36 | 21 | 20 | 1.05 | 1.11 | 0.95 | 0.49 | 1.84 |
| Clotiazepam | 32,494 | 86 | 7 | 6 | 10 | 10 | 1.00 | 1.05 | 0.95 | 0.36 | 2.55 |
|  |  |  |  | 12 | 12 | 14 | 0.86 | 1.08 | 0.80 | 0.34 | 1.85 |
|  |  |  |  | 24 | 22 | 22 | 1.00 | 1.14 | 0.88 | 0.46 | 1.66 |
|  |  |  |  | 36 | 26 | 28 | 0.93 | 1.19 | 0.78 | 0.44 | 1.38 |
| Bromazepam | 12,281 | 40 | 1 | 6 | 7 | 4 | 1.75 | 1.05 | 1.67 | 0.42 | 7.76 |
|  |  |  |  | 12 | 13 | 5 | 2.60 | 1.08 | 2.41 | 0.81 | 8.64 |
|  |  |  |  | 24 | 17 | 11 | 1.55 | 1.13 | 1.37 | 0.61 | 3.23 |
|  |  |  |  | 36 | 19 | 14 | 1.36 | 1.17 | 1.16 | 0.55 | 2.50 |
| Hydroxyzine | 66,670 | 328 | 64 | 6 | 47 | 87 | 0.54 | 1.04 | 0.52 | 0.36 | 0.75 |
|  |  |  |  | 12 | 66 | 106 | 0.62 | 1.07 | 0.58 | 0.42 | 0.80 |
|  |  |  |  | 24 | 80 | 122 | 0.66 | 1.12 | 0.59 | 0.44 | 0.78 |
|  |  |  |  | 36 | 93 | 131 | 0.71 | 1.16 | 0.61 | 0.47 | 0.81 |
| Cloxazolam | 4,937 | 37 | 5 | 6 | 2 | 12 | 0.17 | 1.05 | 0.16 | 0.02 | 0.71 |
|  |  |  |  | 12 | 4 | 16 | 0.25 | 1.09 | 0.23 | 0.06 | 0.71 |
|  |  |  |  | 24 | 4 | 20 | 0.20 | 1.16 | 0.17 | 0.04 | 0.51 |
|  |  |  |  | 36 | 5 | 23 | 0.22 | 1.22 | 0.18 | 0.05 | 0.48 |
| Dandospirone | 7,114 | 35 | 3 | 6 | 7 | 5 | 1.40 | 1.04 | 1.34 | 0.37 | 5.36 |
|  |  |  |  | 12 | 7 | 7 | 1.00 | 1.07 | 0.94 | 0.28 | 3.14 |
|  |  |  |  | 24 | 10 | 14 | 0.71 | 1.11 | 0.64 | 0.26 | 1.56 |
|  |  |  |  | 36 | 13 | 14 | 0.93 | 1.15 | 0.81 | 0.35 | 1.86 |
| Tofisopam | 10,014 | 26 | 0 | 6 | 2 | 4 | 0.50 | 1.05 | 0.48 | 0.04 | 3.34 |
|  |  |  |  | 12 | 5 | 6 | 0.83 | 1.07 | 0.78 | 0.19 | 3.06 |
|  |  |  |  | 24 | 6 | 10 | 0.60 | 1.12 | 0.54 | 0.16 | 1.63 |
|  |  |  |  | 36 | 9 | 10 | 0.90 | 1.17 | 0.77 | 0.28 | 2.11 |
