# Supplementary material for: An integrative approach using real-world data to identify alternative therapeutic uses of existing drugs
Source: PLoS One. 2018 Oct 9;13(10):e0204648. doi: 10.1371/journal.pone.0204648 (PMC6177143; doi:10.1371/journal.pone.0204648)
Supplement: S5 Table — Inverse associations were detected for haloperidol at least three intervals. (DOCX) [file pone.0204648.s005.docx]

S5 Table. Association between psycholeptics (N05A) and ulcerative colitis (JMDC claims database)

Inverse associations were detected for haloperidol at least three intervals.

|  | Incident users | Cocomitant users | Simultaneous start | interval (months) | last | first | Crude SR | Null-Effect SR | Adjusted SR | 95%CI | |
| --- | --- | --- | --- | --- | --- | --- | --- | --- | --- | --- | --- |
|  |  |  |  |  |  |  |  |  |  | Lower | Upper |
| Risperidone | 10,819 | 77 | 6 | 6 | 5 | 14 | 0.36 | 0.99 | 0.36 | 0.10 | 1.06 |
|  |  |  |  | 12 | 7 | 22 | 0.32 | 1.00 | 0.32 | 0.12 | 0.77 |
|  |  |  |  | 24 | 15 | 29 | 0.52 | 1.01 | 0.51 | 0.26 | 0.99 |
|  |  |  |  | 36 | 23 | 34 | 0.68 | 1.03 | 0.66 | 0.37 | 1.15 |
| Aripiprazole | 14,186 | 99 | 2 | 6 | 11 | 15 | 0.73 | 0.98 | 0.75 | 0.31 | 1.74 |
|  |  |  |  | 12 | 15 | 26 | 0.58 | 0.97 | 0.59 | 0.29 | 1.16 |
|  |  |  |  | 24 | 23 | 37 | 0.62 | 0.95 | 0.66 | 0.37 | 1.13 |
|  |  |  |  | 36 | 32 | 44 | 0.73 | 0.91 | 0.80 | 0.49 | 1.29 |
| Olanzapine | 9,905 | 82 | 2 | 6 | 9 | 13 | 0.69 | 1.01 | 0.68 | 0.26 | 1.73 |
|  |  |  |  | 12 | 17 | 21 | 0.81 | 1.03 | 0.79 | 0.39 | 1.56 |
|  |  |  |  | 24 | 24 | 28 | 0.86 | 1.06 | 0.80 | 0.45 | 1.44 |
|  |  |  |  | 36 | 28 | 34 | 0.82 | 1.08 | 0.76 | 0.44 | 1.29 |
| Quetiapine | 6,880 | 57 | 1 | 6 | 8 | 6 | 1.33 | 1.01 | 1.32 | 0.40 | 4.63 |
|  |  |  |  | 12 | 13 | 15 | 0.87 | 1.02 | 0.85 | 0.37 | 1.92 |
|  |  |  |  | 24 | 16 | 19 | 0.84 | 1.03 | 0.82 | 0.39 | 1.68 |
|  |  |  |  | 36 | 20 | 24 | 0.83 | 1.04 | 0.80 | 0.42 | 1.51 |
| Levomepromazine | 3,889 | 27 | 0 | 6 | 1 | 2 | 0.50 | 1.02 | 0.49 | 0.01 | 9.43 |
|  |  |  |  | 12 | 1 | 6 | 0.17 | 1.04 | 0.16 | 0.00 | 1.32 |
|  |  |  |  | 24 | 5 | 11 | 0.45 | 1.07 | 0.43 | 0.12 | 1.33 |
|  |  |  |  | 36 | 8 | 13 | 0.62 | 1.08 | 0.57 | 0.20 | 1.48 |
| Haloperidol | 6,797 | 58 | 5 | 6 | 7 | 16 | 0.44 | 1.01 | 0.44 | 0.15 | 1.12 |
|  |  |  |  | 12 | 8 | 23 | 0.35 | 1.01 | 0.34 | 0.13 | 0.80 |
|  |  |  |  | 24 | 9 | 29 | 0.31 | 1.02 | 0.30 | 0.13 | 0.66 |
|  |  |  |  | 36 | 10 | 33 | 0.30 | 1.03 | 0.29 | 0.13 | 0.61 |
| Chlorpromazine | 4,000 | 30 | 1 | 6 | 1 | 8 | 0.13 | 0.99 | 0.13 | 0.00 | 0.94 |
|  |  |  |  | 12 | 3 | 11 | 0.27 | 1.00 | 0.27 | 0.05 | 1.03 |
|  |  |  |  | 24 | 7 | 14 | 0.50 | 1.03 | 0.49 | 0.17 | 1.29 |
|  |  |  |  | 36 | 8 | 16 | 0.50 | 1.05 | 0.48 | 0.18 | 1.18 |
| Blonaserin | 2,103 | 20 | 0 | 6 | 1 | 5 | 0.20 | 1.01 | 0.20 | 0.00 | 1.77 |
|  |  |  |  | 12 | 2 | 6 | 0.33 | 1.02 | 0.33 | 0.03 | 1.82 |
|  |  |  |  | 24 | 6 | 7 | 0.86 | 1.05 | 0.82 | 0.23 | 2.84 |
|  |  |  |  | 36 | 7 | 9 | 0.78 | 1.07 | 0.73 | 0.23 | 2.20 |
| Perospirone | 1,900 | 18 | 1 | 6 | 0 | 2 | 0.00 | 1.00 | 0.00 | - | - |
|  |  |  |  | 12 | 2 | 6 | 0.33 | 1.02 | 0.33 | 0.03 | 1.83 |
|  |  |  |  | 24 | 4 | 6 | 0.67 | 1.05 | 0.64 | 0.13 | 2.68 |
|  |  |  |  | 36 | 6 | 7 | 0.86 | 1.07 | 0.80 | 0.22 | 2.77 |
| Zotepine | 612 | 1 | 0 | 6 | 0 | 0 | - | 1.03 | - | - | - |
|  |  |  |  | 12 | 0 | 0 | - | 1.06 | - | - | - |
|  |  |  |  | 24 | 0 | 0 | - | 1.12 | - | - | - |
|  |  |  |  | 36 | 0 | 0 | - | 1.16 | - | - | - |
| Sulpiride | 2,251 | 18 | 1 | 6 | 1 | 2 | 0.50 | 1.00 | 0.50 | 0.01 | 9.64 |
|  |  |  |  | 12 | 3 | 3 | 1.00 | 1.00 | 1.00 | 0.13 | 7.44 |
|  |  |  |  | 24 | 6 | 5 | 1.20 | 1.03 | 1.17 | 0.30 | 4.84 |
|  |  |  |  | 36 | 6 | 5 | 1.20 | 1.05 | 1.14 | 0.29 | 4.72 |
| Prochlorperazine | 7,197 | 52 | 5 | 6 | 5 | 15 | 0.33 | 0.97 | 0.34 | 0.10 | 0.99 |
|  |  |  |  | 12 | 11 | 20 | 0.55 | 0.97 | 0.57 | 0.25 | 1.24 |
|  |  |  |  | 24 | 15 | 24 | 0.63 | 0.95 | 0.66 | 0.32 | 1.31 |
|  |  |  |  | 36 | 15 | 27 | 0.56 | 0.93 | 0.60 | 0.30 | 1.16 |
| Paliperidone | 910 | 5 | 0 | 6 | 0 | 0 | - | 1.06 | - | - | - |
|  |  |  |  | 12 | 0 | 1 | 0.00 | 1.12 | 0.00 | - | - |
|  |  |  |  | 24 | 1 | 2 | 0.50 | 1.23 | 0.41 | 0.01 | 7.80 |
|  |  |  |  | 36 | 2 | 2 | 1.00 | 1.31 | 0.77 | 0.06 | 10.57 |
| Bromperidol | 299 | 5 | 0 | 6 | 0 | 0 | - | 1.07 | - | ‐ | ‐ |
|  |  |  |  | 12 | 0 | 0 | - | 1.15 | - | ‐ | ‐ |
|  |  |  |  | 24 | 0 | 1 | 0.00 | 1.28 | 0.00 | ‐ | ‐ |
|  |  |  |  | 36 | 1 | 1 | 1.00 | 1.33 | 0.75 | 0.01 | 59.17 |
| Perphenazine | 1,162 | 10 | 0 | 6 | 0 | 1 | 0.00 | 0.99 | 0.00 | - | - |
|  |  |  |  | 12 | 1 | 2 | 0.50 | 1.01 | 0.50 | 0.01 | 9.52 |
|  |  |  |  | 24 | 2 | 3 | 0.67 | 1.05 | 0.64 | 0.05 | 5.54 |
|  |  |  |  | 36 | 2 | 4 | 0.50 | 1.09 | 0.46 | 0.04 | 3.21 |
| Propericiazine | 348 | 1 | 0 | 6 | 0 | 0 | - | 1.02 | - | ‐ | ‐ |
|  |  |  |  | 12 | 0 | 0 | - | 1.05 | - | ‐ | ‐ |
|  |  |  |  | 24 | 0 | 1 | 0.00 | 1.11 | 0.00 | ‐ | ‐ |
|  |  |  |  | 36 | 0 | 1 | 0.00 | 1.14 | 0.00 | ‐ | ‐ |
| Tiapride | 533 | 3 | 0 | 6 | 1 | 0 | - | 0.98 | - | ‐ | ‐ |
|  |  |  |  | 12 | 1 | 0 | - | 0.98 | - | ‐ | ‐ |
|  |  |  |  | 24 | 1 | 1 | 1.00 | 0.99 | 1.01 | 0.01 | 79.16 |
|  |  |  |  | 36 | 1 | 1 | 1.00 | 1.02 | 0.98 | 0.01 | 76.70 |
