# Supplementary material for: An integrative approach using real-world data to identify alternative therapeutic uses of existing drugs
Source: PLoS One. 2018 Oct 9;13(10):e0204648. doi: 10.1371/journal.pone.0204648 (PMC6177143; doi:10.1371/journal.pone.0204648)
Supplement: S6 Table — Inverse associations were detected for zolpidem, flunitrazepam, zopiclone at least three intervals. (DOCX) [file pone.0204648.s006.docx]

S6 Table. Association between psycholeptics (N05B) and ulcerative colitis (JMDC claims database)

Inverse associations were detected for zolpidem, flunitrazepam, zopiclone at least three intervals.

| Drugs | Incident users | Cocomitant users | Simultaneous start | interval (months) | last | first | Crude SR | Null-Effect SR | Adjusted SR | 95%CI | |
| --- | --- | --- | --- | --- | --- | --- | --- | --- | --- | --- | --- |
|  |  |  |  |  |  |  |  |  |  | Lower | Upper |
| Ramelteon | 10,690 | 82 | 3 | 6 | 11 | 11 | 1.00 | 0.99 | 1.01 | 0.40 | 2.56 |
|  |  |  |  | 12 | 19 | 19 | 1.00 | 0.98 | 1.02 | 0.51 | 2.03 |
|  |  |  |  | 24 | 23 | 28 | 0.82 | 0.97 | 0.85 | 0.47 | 1.53 |
|  |  |  |  | 36 | 30 | 31 | 0.97 | 0.95 | 1.01 | 0.59 | 1.73 |
| Brotizolam | 54,573 | 422 | 28 | 6 | 36 | 66 | 0.55 | 1.02 | 0.54 | 0.35 | 0.82 |
|  |  |  |  | 12 | 71 | 94 | 0.76 | 1.03 | 0.73 | 0.53 | 1.01 |
|  |  |  |  | 24 | 117 | 132 | 0.89 | 1.06 | 0.83 | 0.65 | 1.08 |
|  |  |  |  | 36 | 148 | 154 | 0.96 | 1.08 | 0.89 | 0.70 | 1.12 |
| Zolpidem | 66,544 | 507 | 36 | 6 | 55 | 81 | 0.68 | 1.01 | 0.67 | 0.47 | 0.96 |
|  |  |  |  | 12 | 89 | 111 | 0.80 | 1.02 | 0.78 | 0.59 | 1.04 |
|  |  |  |  | 24 | 138 | 175 | 0.79 | 1.05 | 0.75 | 0.60 | 0.94 |
|  |  |  |  | 36 | 164 | 201 | 0.82 | 1.07 | 0.76 | 0.62 | 0.94 |
| Flunitrazepam | 45,783 | 850 | 329 | 6 | 55 | 153 | 0.36 | 1.01 | 0.36 | 0.26 | 0.49 |
|  |  |  |  | 12 | 82 | 189 | 0.43 | 1.02 | 0.43 | 0.33 | 0.56 |
|  |  |  |  | 24 | 138 | 234 | 0.59 | 1.04 | 0.57 | 0.46 | 0.71 |
|  |  |  |  | 36 | 167 | 254 | 0.66 | 1.05 | 0.63 | 0.51 | 0.76 |
| Triazolam | 17,893 | 138 | 4 | 6 | 21 | 21 | 1.00 | 1.03 | 0.97 | 0.50 | 1.86 |
|  |  |  |  | 12 | 27 | 33 | 0.82 | 1.07 | 0.77 | 0.44 | 1.32 |
|  |  |  |  | 24 | 47 | 45 | 1.04 | 1.13 | 0.92 | 0.60 | 1.42 |
|  |  |  |  | 36 | 53 | 53 | 1.00 | 1.19 | 0.84 | 0.56 | 1.26 |
| Nitrazepam | 8,932 | 60 | 1 | 6 | 5 | 6 | 0.83 | 1.02 | 0.82 | 0.20 | 3.21 |
|  |  |  |  | 12 | 11 | 9 | 1.22 | 1.05 | 1.17 | 0.44 | 3.19 |
|  |  |  |  | 24 | 16 | 23 | 0.70 | 1.10 | 0.63 | 0.31 | 1.25 |
|  |  |  |  | 36 | 20 | 25 | 0.80 | 1.15 | 0.70 | 0.37 | 1.31 |
| Zopiclone | 14,366 | 112 | 3 | 6 | 10 | 25 | 0.40 | 1.04 | 0.39 | 0.17 | 0.83 |
|  |  |  |  | 12 | 19 | 34 | 0.56 | 1.07 | 0.52 | 0.28 | 0.94 |
|  |  |  |  | 24 | 30 | 46 | 0.65 | 1.14 | 0.57 | 0.35 | 0.93 |
|  |  |  |  | 36 | 34 | 54 | 0.63 | 1.20 | 0.52 | 0.33 | 0.82 |
| Estazolam | 8,499 | 89 | 4 | 6 | 5 | 10 | 0.50 | 1.03 | 0.49 | 0.13 | 1.56 |
|  |  |  |  | 12 | 14 | 15 | 0.93 | 1.06 | 0.88 | 0.40 | 1.96 |
|  |  |  |  | 24 | 24 | 25 | 0.96 | 1.12 | 0.85 | 0.47 | 1.56 |
|  |  |  |  | 36 | 27 | 29 | 0.93 | 1.19 | 0.78 | 0.45 | 1.37 |
| Rilmazafone | 11,849 | 94 | 5 | 6 | 5 | 19 | 0.26 | 1.02 | 0.26 | 0.08 | 0.71 |
|  |  |  |  | 12 | 10 | 25 | 0.40 | 1.05 | 0.38 | 0.16 | 0.82 |
|  |  |  |  | 24 | 23 | 36 | 0.64 | 1.11 | 0.58 | 0.33 | 1.00 |
|  |  |  |  | 36 | 32 | 39 | 0.82 | 1.16 | 0.71 | 0.43 | 1.16 |
| Eszopiclone | 11,852 | 97 | 4 | 6 | 11 | 16 | 0.69 | 0.95 | 0.73 | 0.31 | 1.67 |
|  |  |  |  | 12 | 15 | 29 | 0.52 | 0.89 | 0.58 | 0.29 | 1.11 |
|  |  |  |  | 24 | 23 | 42 | 0.55 | 0.80 | 0.69 | 0.39 | 1.17 |
|  |  |  |  | 36 | 25 | 46 | 0.54 | 0.70 | 0.78 | 0.46 | 1.30 |
| Lormetazepam | 4,938 | 35 | 1 | 6 | 4 | 6 | 0.67 | 1.03 | 0.65 | 0.13 | 2.73 |
|  |  |  |  | 12 | 7 | 11 | 0.64 | 1.05 | 0.61 | 0.20 | 1.71 |
|  |  |  |  | 24 | 12 | 15 | 0.80 | 1.09 | 0.73 | 0.31 | 1.68 |
|  |  |  |  | 36 | 12 | 19 | 0.63 | 1.13 | 0.56 | 0.25 | 1.21 |
| Phenobarbital | 4,871 | 10 | 1 | 6 | 1 | 1 | 1.00 | 1.04 | 0.96 | 0.01 | 75.14 |
|  |  |  |  | 12 | 1 | 1 | 1.00 | 1.10 | 0.91 | 0.01 | 71.24 |
|  |  |  |  | 24 | 3 | 1 | 3.00 | 1.19 | 2.52 | 0.20 | 132.46 |
|  |  |  |  | 36 | 5 | 1 | 5.00 | 1.26 | 3.98 | 0.45 | 188.06 |
| Quazepam | 3,769 | 37 | 0 | 6 | 2 | 6 | 0.33 | 1.06 | 0.31 | 0.03 | 1.76 |
|  |  |  |  | 12 | 3 | 9 | 0.33 | 1.11 | 0.30 | 0.05 | 1.20 |
|  |  |  |  | 24 | 6 | 16 | 0.38 | 1.20 | 0.31 | 0.10 | 0.84 |
|  |  |  |  | 36 | 11 | 17 | 0.65 | 1.28 | 0.51 | 0.21 | 1.15 |
| Triclofos | 13,847 | 12 | 3 | 6 | 3 | 0 | - | 1.02 | - | - | - |
|  |  |  |  | 12 | 3 | 0 | - | 1.04 | - | - | - |
|  |  |  |  | 24 | 3 | 0 | - | 1.07 | - | - | - |
|  |  |  |  | 36 | 4 | 0 | - | 1.10 | - | - | - |
| Suvorexant | 5,785 | 39 | 1 | 6 | 9 | 3 | 3.00 | 0.69 | 4.34 | 1.08 | 24.94 |
|  |  |  |  | 12 | 15 | 7 | 2.14 | 0.51 | 4.19 | 1.61 | 12.15 |
|  |  |  |  | 24 | 15 | 13 | 1.15 | 0.29 | 4.02 | 1.79 | 9.18 |
|  |  |  |  | 36 | 15 | 15 | 1.00 | 0.22 | 4.56 | 2.08 | 10.02 |
| Flurazepam | 628 | 3 | 0 | 6 | 0 | 0 | - | 1.06 | - | - | - |
|  |  |  |  | 12 | 0 | 0 | - | 1.12 | - | - | - |
|  |  |  |  | 24 | 0 | 0 | - | 1.23 | - | - | - |
|  |  |  |  | 36 | 1 | 0 | - | 1.31 | - | - | - |
| Bromovalerylurea | 4,169 | 21 | 2 | 6 | 2 | 1 | 2.00 | 1.01 | 1.99 | 0.10 | 117.36 |
|  |  |  |  | 12 | 4 | 3 | 1.33 | 1.05 | 1.27 | 0.22 | 8.66 |
|  |  |  |  | 24 | 5 | 3 | 1.67 | 1.13 | 1.47 | 0.29 | 9.48 |
|  |  |  |  | 36 | 7 | 4 | 1.75 | 1.21 | 1.44 | 0.37 | 6.72 |
| Nimetazepam | 777 | 11 | 1 | 6 | 0 | 1 | 0.00 | 1.11 | 0.00 | - | - |
|  |  |  |  | 12 | 0 | 2 | 0.00 | 1.18 | 0.00 | - | - |
|  |  |  |  | 24 | 3 | 2 | 1.50 | 1.28 | 1.17 | 0.13 | 13.98 |
|  |  |  |  | 36 | 4 | 2 | 2.00 | 1.36 | 1.47 | 0.21 | 16.29 |
| Amobarbital | 360 | 3 | 0 | 6 | 0 | 0 | - | 1.04 | - | - | - |
|  |  |  |  | 12 | 0 | 0 | - | 1.09 | - | - | - |
|  |  |  |  | 24 | 1 | 0 | - | 1.20 | - | - | - |
|  |  |  |  | 36 | 1 | 0 | - | 1.30 | - | - | - |
| Chloral hydrate | 5,321 | 7 | 0 | 6 | 3 | 0 | - | 1.02 | - | - | - |
|  |  |  |  | 12 | 3 | 0 | - | 1.05 | - | - | - |
|  |  |  |  | 24 | 3 | 0 | - | 1.11 | - | - | - |
|  |  |  |  | 36 | 4 | 0 | - | 1.15 | - | - | - |
| Haloxazolam | 407 | 4 | 0 | 6 | 0 | 0 | - | 1.04 | - | - | - |
|  |  |  |  | 12 | 0 | 0 | - | 1.07 | - | - | - |
|  |  |  |  | 24 | 1 | 1 | 1.00 | 1.15 | 0.87 | 0.01 | 68.41 |
|  |  |  |  | 36 | 2 | 1 | 2.00 | 1.25 | 1.60 | 0.08 | 94.17 |
