# Supplementary material for: An integrative approach using real-world data to identify alternative therapeutic uses of existing drugs
Source: PLoS One. 2018 Oct 9;13(10):e0204648. doi: 10.1371/journal.pone.0204648 (PMC6177143; doi:10.1371/journal.pone.0204648)
Supplement: S7 Table — Inverse associations were detected for diazepam and hydroxyzine at least three intervals. (DOCX) [file pone.0204648.s007.docx]

S7 Table. Association between psycholeptics (N05C) and ulcerative colitis (JMDC claims database)

Inverse associations were detected for diazepam and hydroxyzine at least three intervals.

| Drugs | Incident users | Cocomitant users | Simultaneous start | interval (months) | last | first | Crude SR | Null-Effect SR | Adjusted SR | 95%CI | |
| --- | --- | --- | --- | --- | --- | --- | --- | --- | --- | --- | --- |
|  |  |  |  |  |  |  |  |  |  | Lower | Upper |
| Etizolam | 76,230 | 594 | 38 | 6 | 58 | 75 | 0.77 | 1.02 | 0.76 | 0.53 | 1.08 |
|  |  |  |  | 12 | 114 | 114 | 1.00 | 1.04 | 0.96 | 0.73 | 1.25 |
|  |  |  |  | 24 | 174 | 166 | 1.05 | 1.09 | 0.96 | 0.78 | 1.20 |
|  |  |  |  | 36 | 213 | 209 | 1.02 | 1.12 | 0.91 | 0.75 | 1.11 |
| Alprazolam | 33,417 | 279 | 13 | 6 | 27 | 42 | 0.64 | 1.02 | 0.63 | 0.37 | 1.05 |
|  |  |  |  | 12 | 47 | 66 | 0.71 | 1.04 | 0.69 | 0.46 | 1.01 |
|  |  |  |  | 24 | 74 | 102 | 0.73 | 1.07 | 0.68 | 0.50 | 0.92 |
|  |  |  |  | 36 | 92 | 118 | 0.78 | 1.10 | 0.71 | 0.53 | 0.94 |
| Ethyl loflazepate | 29,299 | 266 | 9 | 6 | 28 | 31 | 0.90 | 1.02 | 0.89 | 0.51 | 1.53 |
|  |  |  |  | 12 | 53 | 47 | 1.13 | 1.03 | 1.09 | 0.72 | 1.65 |
|  |  |  |  | 24 | 80 | 70 | 1.14 | 1.07 | 1.07 | 0.76 | 1.49 |
|  |  |  |  | 36 | 106 | 93 | 1.14 | 1.11 | 1.03 | 0.77 | 1.37 |
| Diazepam | 84,620 | 1030 | 352 | 6 | 99 | 173 | 0.57 | 1.01 | 0.57 | 0.44 | 0.73 |
|  |  |  |  | 12 | 151 | 221 | 0.68 | 1.03 | 0.67 | 0.54 | 0.82 |
|  |  |  |  | 24 | 211 | 290 | 0.73 | 1.05 | 0.69 | 0.58 | 0.83 |
|  |  |  |  | 36 | 243 | 324 | 0.75 | 1.07 | 0.70 | 0.59 | 0.83 |
| Lorazepam | 20,320 | 165 | 4 | 6 | 23 | 26 | 0.88 | 1.01 | 0.88 | 0.48 | 1.60 |
|  |  |  |  | 12 | 33 | 37 | 0.89 | 1.01 | 0.88 | 0.53 | 1.45 |
|  |  |  |  | 24 | 58 | 50 | 1.16 | 1.03 | 1.13 | 0.76 | 1.69 |
|  |  |  |  | 36 | 66 | 59 | 1.12 | 1.03 | 1.08 | 0.75 | 1.56 |
| Clotiazepam | 32,494 | 299 | 14 | 6 | 30 | 30 | 1.00 | 1.02 | 0.98 | 0.57 | 1.68 |
|  |  |  |  | 12 | 52 | 58 | 0.90 | 1.04 | 0.86 | 0.58 | 1.28 |
|  |  |  |  | 24 | 84 | 95 | 0.88 | 1.08 | 0.82 | 0.60 | 1.11 |
|  |  |  |  | 36 | 103 | 109 | 0.94 | 1.12 | 0.84 | 0.64 | 1.11 |
| Bromazepam | 12,281 | 103 | 3 | 6 | 12 | 20 | 0.60 | 1.02 | 0.59 | 0.26 | 1.26 |
|  |  |  |  | 12 | 16 | 30 | 0.53 | 1.04 | 0.52 | 0.26 | 0.98 |
|  |  |  |  | 24 | 29 | 38 | 0.76 | 1.07 | 0.72 | 0.43 | 1.19 |
|  |  |  |  | 36 | 34 | 48 | 0.71 | 1.10 | 0.65 | 0.40 | 1.02 |
| Hydroxyzine | 66,670 | 531 | 92 | 6 | 56 | 97 | 0.58 | 1.01 | 0.57 | 0.40 | 0.80 |
|  |  |  |  | 12 | 89 | 131 | 0.68 | 1.03 | 0.66 | 0.50 | 0.87 |
|  |  |  |  | 24 | 123 | 168 | 0.73 | 1.06 | 0.69 | 0.54 | 0.88 |
|  |  |  |  | 36 | 145 | 198 | 0.73 | 1.09 | 0.67 | 0.54 | 0.84 |
| Cloxazolam | 4,937 | 48 | 2 | 6 | 10 | 4 | 2.50 | 1.02 | 2.45 | 0.71 | 10.69 |
|  |  |  |  | 12 | 16 | 8 | 2.00 | 1.05 | 1.90 | 0.77 | 5.14 |
|  |  |  |  | 24 | 19 | 9 | 2.11 | 1.10 | 1.92 | 0.83 | 4.81 |
|  |  |  |  | 36 | 20 | 14 | 1.43 | 1.15 | 1.24 | 0.60 | 2.66 |
| Dandospirone | 7,114 | 76 | 9 | 6 | 14 | 7 | 2.00 | 1.01 | 1.97 | 0.75 | 5.78 |
|  |  |  |  | 12 | 19 | 9 | 2.11 | 1.02 | 2.06 | 0.89 | 5.18 |
|  |  |  |  | 24 | 28 | 16 | 1.75 | 1.05 | 1.67 | 0.87 | 3.30 |
|  |  |  |  | 36 | 36 | 20 | 1.80 | 1.07 | 1.68 | 0.95 | 3.06 |
| Tofisopam | 10,014 | 97 | 5 | 6 | 9 | 11 | 0.82 | 1.02 | 0.80 | 0.29 | 2.13 |
|  |  |  |  | 12 | 20 | 20 | 1.00 | 1.03 | 0.97 | 0.50 | 1.90 |
|  |  |  |  | 24 | 29 | 34 | 0.85 | 1.06 | 0.80 | 0.47 | 1.36 |
|  |  |  |  | 36 | 35 | 40 | 0.88 | 1.10 | 0.80 | 0.49 | 1.29 |
