# Supplementary material for: An integrative approach using real-world data to identify alternative therapeutic uses of existing drugs
Source: PLoS One. 2018 Oct 9;13(10):e0204648. doi: 10.1371/journal.pone.0204648 (PMC6177143; doi:10.1371/journal.pone.0204648)
Supplement: S8 Table — Haloperidol, estazolam, rilmazafone, diazepam, hydroxyzine, and cloxazolam were inversely associated with a diagnosis of CD; and haloperidol, zolpidem, flunitrazepam, zopiclone, diazepam, and hydroxyzine were inversely associated with a diagnosis of UC. (DOCX) [file pone.0204648.s008.docx]

S8 Table. Summary of sequence symmetry analyses (JMDC claims database)

Haloperidol, estazolam, rilmazafone, diazepam, hydroxyzine, and cloxazolam were inversely associated with a diagnosis of CD; and haloperidol, zolpidem, flunitrazepam, zopiclone, diazepam, and hydroxyzine were inversely associated with a diagnosis of UC.

|  |  | Crohn's disease (K50) | | | | Ulcerative colitis (K51) | | | |
| --- | --- | --- | --- | --- | --- | --- | --- | --- | --- |
|  |  | Interval (months) | | | | Interval (months) | | | |
| ATC code | Drugs | 6 | 12 | 24 | 36 | 6 | 12 | 24 | 36 |
| N05A | Risperidone | ‐ | ‐ | ‐ | ‐ | - | ▼ | ▼ | ‐ |
|  | Aripiprazole | ‐ | ‐ | ‐ | ‐ | ‐ | ‐ | ‐ | ‐ |
|  | Olanzapine | ‐ | ‐ | ‐ | ‐ | ‐ | ‐ | ‐ | ‐ |
|  | Quetiapine | ‐ | ‐ | ‐ | ‐ | ‐ | ‐ | ‐ | ‐ |
|  | Levomepromazine | ‐ | ‐ | ‐ | ‐ | ‐ | ‐ | ‐ | ‐ |
|  | Haloperidol | ▼ | ▼ | ▼ | ▼ | ‐ | ▼ | ▼ | ▼ |
|  | Chlorpromazine | ‐ | ‐ | ‐ | ‐ | ▼ | - | ‐ | ‐ |
|  | Blonaserin | ‐ | ‐ | ‐ | ‐ | ‐ | ‐ | ‐ | ‐ |
|  | Perospirone | ‐ | ‐ | ‐ | ‐ | ‐ | ‐ | ‐ | ‐ |
|  | Zotepine | ‐ | ‐ | ‐ | ‐ | ‐ | ‐ | ‐ | ‐ |
|  | Sulpiride | ‐ | ‐ | ‐ | ‐ | ‐ | ‐ | ‐ | ‐ |
|  | Prochlorperazine | ‐ | ‐ | ‐ | ‐ | ▼ | ‐ | ‐ | ‐ |
|  | Paliperidone | ‐ | ‐ | ‐ | ‐ | ‐ | ‐ | ‐ | ‐ |
|  | Bromperidol | ‐ | ‐ | ‐ | ‐ | ‐ | ‐ | ‐ | ‐ |
|  | Perphenazine | ‐ | ‐ | ‐ | ‐ | ‐ | ‐ | ‐ | ‐ |
|  | Propericiazine | ‐ | ‐ | ‐ | ‐ | ‐ | ‐ | ‐ | ‐ |
|  | Tiapride | ‐ | ‐ | ‐ | ‐ | ‐ | ‐ | ‐ | ‐ |
| N05B | Ramelteon | ‐ | ‐ | ‐ | ‐ | ‐ | ‐ | ‐ | ‐ |
|  | Brotizolam | ‐ | ‐ | ‐ | ‐ | ▼ | - | ‐ | ‐ |
|  | Zolpidem | ‐ | ‐ | ‐ | ‐ | ▼ | ‐ | ▼ | ▼ |
|  | Flunitrazepam | - | ‐ | ‐ | ‐ | ▼ | ▼ | ▼ | ▼ |
|  | Triazolam | ‐ | ‐ | ‐ | ‐ | ‐ | ‐ | ‐ | ‐ |
|  | Nitrazepam | ‐ | ‐ | ‐ | ‐ | ‐ | ‐ | ‐ | ‐ |
|  | Zopiclone | ‐ | ‐ | ‐ | ‐ | ▼ | ▼ | ▼ | ▼ |
|  | Estazolam | ▼ | ▼ | ▼ | ▼ | ‐ | ‐ | ‐ | ‐ |
|  | Rilmazafone | ▼ | ▼ | ▼ | ▼ | ▼ | ▼ | - | ‐ |
|  | Eszopiclone | ‐ | ‐ | ‐ | ‐ | ‐ | ‐ | ‐ | ‐ |
|  | Lormetazepam | ‐ | ‐ | ‐ | ‐ | ‐ | ‐ | ‐ | ‐ |
|  | Phenobarbital | ‐ | ‐ | ‐ | ‐ | ‐ | ‐ | ‐ | ‐ |
|  | Quazepam | ‐ | ‐ | ‐ | ‐ | ‐ | ‐ | ▼ | ‐ |
|  | Triclofos | ‐ | ‐ | ‐ | ‐ | ‐ | ‐ | ‐ | ‐ |
|  | Suvorexant | ‐ | ‐ | ‐ | ‐ | △ | △ | △ | △ |
|  | Flurazepam | ‐ | ‐ | ‐ | ‐ | ‐ | ‐ | ‐ | ‐ |
|  | Bromovalerylurea | ‐ | ‐ | ‐ | ‐ | ‐ | ‐ | ‐ | ‐ |
|  | Nimetazepam | ‐ | ‐ | ‐ | ‐ | ‐ | ‐ | ‐ | ‐ |
|  | Amobarbital | ‐ | ‐ | ‐ | ‐ | ‐ | ‐ | ‐ | ‐ |
|  | Chloral hydrate | ‐ | ‐ | ‐ | ‐ | ‐ | ‐ | ‐ | ‐ |
|  | Haloxazolam | ‐ | ‐ | ‐ | ‐ | ‐ | ‐ | ‐ | ‐ |
| N05C | Etizolam | ‐ | ‐ | ‐ | ‐ | ‐ | ‐ | ‐ | ‐ |
|  | Alprazolam | ‐ | ‐ | ‐ | ‐ | ‐ | ‐ | ▼ | ▼ |
|  | Ethyl loflazepate | ‐ | ‐ | ‐ | ▼ | ‐ | ‐ | ‐ | ‐ |
|  | Diazepam | ▼ | ▼ | ▼ | ▼ | ▼ | ▼ | ▼ | ▼ |
|  | Lorazepam | ‐ | ‐ | ‐ | ‐ | ‐ | ‐ | ‐ | ‐ |
|  | Clotiazepam | ‐ | ‐ | ‐ | ‐ | ‐ | ‐ | ‐ | ‐ |
|  | Bromazepam | ‐ | ‐ | ‐ | ‐ | ‐ | ▼ | ‐ | ‐ |
|  | Hydroxyzine | ▼ | ▼ | ▼ | ▼ | ▼ | ▼ | ▼ | ▼ |
|  | Hydroxyzine pamoate | ‐ | ‐ | ‐ | ‐ | ‐ | ‐ | ‐ | ‐ |
|  | Cloxazolam | ▼ | ▼ | ▼ | ▼ | ‐ | ‐ | ‐ | ‐ |
|  | Dandospirone | ‐ | ‐ | ‐ | ‐ | ‐ | ‐ | ‐ | ‐ |
|  | Tofisopam | ‐ | ‐ | ‐ | ‐ | ‐ | ‐ | ‐ | ‐ |
