# Supplementary material for: An integrative approach using real-world data to identify alternative therapeutic uses of existing drugs
Source: PLoS One. 2018 Oct 9;13(10):e0204648. doi: 10.1371/journal.pone.0204648 (PMC6177143; doi:10.1371/journal.pone.0204648)
Supplement: S9 Table — Risperidone, aripiprazole, olanzapine, quetiapine, levomepromazine, haloperidol, chlorpromazine, sulpiride, prochlorperazine, paliperidone, brotizolam, zolpidem, flunitrazepam, triazolam, zopiclone, eszopiclone, phenobarbital, etizolam, diazepam, bromazepam, and hydroxyzine were inversely associated with diagnosis of CD. (DOCX) [file pone.0204648.s009.docx]

S9 Table. Association between psycholeptics and Crohn's disease (FAERS database)

Risperidone, aripiprazole, olanzapine, quetiapine, levomepromazine, haloperidol, chlorpromazine, sulpiride, prochlorperazine, paliperidone, brotizolam, zolpidem, flunitrazepam, triazolam, zopiclone, eszopiclone, phenobarbital, etizolam, diazepam, bromazepam, and hydroxyzine were inversely associated with CD.

| ATC code | Drugs | Crohn's disease (PT: 10011401) | | | |
| --- | --- | --- | --- | --- | --- |
|  |  | Case | Non-cases | ROR ( Lower - Upper ) | IC ( Lower to Upper ) |
| N05A | Risperidone | 26 | 225,993 | 0.16 (0.1-0.23) | -2.59 (-3.14 to -2.03) |
|  | Aripiprazole | 46 | 140,751 | 0.46 (0.34-0.61) | -1.11 (-1.52 to -0.68) |
|  | Olanzapine | 45 | 223,421 | 0.28 (0.21-0.37) | -1.8 (-2.22 to -1.37) |
|  | Quetiapine | 115 | 361,843 | 0.44 (0.37-0.53) | -1.16 (-1.42 to -0.88) |
|  | Levomepromazine | 2 | 20,237 | 0.14 (0.03-0.55) | -2.36 (-4.02 to -0.69) |
|  | Haloperidol | 10 | 101,138 | 0.14 (0.07-0.25) | -2.73 (-3.6 to -1.86) |
|  | Chlorpromazine | 3 | 35,513 | 0.12 (0.03-0.36) | -2.72 (-4.16 to -1.27) |
|  | Blonaserin | 1 | 1,089 | 1.29 (0.18-9.15) | 0.17 (-1.87 to 2.21) |
|  | Perospirone | 0 | 1,566 | - | -1.08 (-3.96 to 1.8) |
|  | Zotepine | 0 | 2,131 | - | -1.33 (-4.21 to 1.55) |
|  | Sulpiride | 1 | 15,457 | 0.09 (0.01-0.64) | -2.59 (-4.62 to -0.54) |
|  | Prochlorperazine | 39 | 131,588 | 0.42 (0.3-0.56) | -1.25 (-1.7 to -0.78) |
|  | Paliperidone | 5 | 53,066 | 0.13 (0.05-0.31) | -2.69 (-3.87 to -1.51) |
|  | Bromperidol | 0 | 785 | - | -0.64 (-3.52 to 2.24) |
|  | Perphenazine | 3 | 12,659 | 0.33 (0.1-1.03) | -1.33 (-2.76 to 0.11) |
|  | Propericiazine | 1 | 1,835 | 0.76 (0.1-5.42) | -0.21 (-2.24 to 1.83) |
|  | Tiapride | 0 | 6,537 | - | -2.5 (-5.38 to 0.38) |
| N05B | Ramelteon | 4 | 14,398 | 0.39 (0.14-1.03) | -1.17 (-2.46 to 0.11) |
|  | Brotizolam | 3 | 30,556 | 0.14 (0.04-0.42) | -2.51 (-3.95 to -1.06) |
|  | Zolpidem | 234 | 411,751 | 0.8 (0.7-0.9) | -0.33 (-0.51 to -0.13) |
|  | Flunitrazepam | 1 | 23,938 | 0.06 (0-0.41) | -3.18 (-5.21 to -1.13) |
|  | Triazolam | 6 | 25,776 | 0.33 (0.14-0.72) | -1.47 (-2.55 to -0.37) |
|  | Nitrazepam | 93 | 34,225 | 3.81 (3.11-4.67) | 1.89 (1.58 to 2.18) |
|  | Zopiclone | 35 | 88,292 | 0.56 (0.39-0.77) | -0.83 (-1.31 to -0.34) |
|  | Estazolam | 0 | 8,509 | - | -2.82 (-5.7 to 0.06) |
|  | Rilmazafone | 1 | 3,735 | 0.38 (0.05-2.66) | -0.87 (-2.91 to 1.16) |
|  | Eszopiclone | 19 | 56,905 | 0.47 (0.29-0.73) | -1.06 (-1.7 to -0.4) |
|  | Lormetazepam | 5 | 11,868 | 0.59 (0.24-1.41) | -0.66 (-1.83 to 0.52) |
|  | Phenobarbital | 23 | 59,354 | 0.54 (0.36-0.81) | -0.85 (-1.44 to -0.26) |
|  | Quazepam | 0 | 3,031 | - | -1.66 (-4.54 to 1.22) |
|  | Triclofos | 0 | 415 | - | -0.38 (-3.26 to 2.51) |
|  | Suvorexant | 0 | 4,417 | - | -2.05 (-4.93 to 0.83) |
|  | Flurazepam | 8 | 14,057 | 0.8 (0.39-1.59) | -0.29 (-1.25 to 0.66) |
|  | Bromovalerylurea | 0 | 331 | - | -0.31 (-3.19 to 2.58) |
|  | Nimetazepam | 0 | 181 | - | -0.18 (-3.06 to 2.71) |
|  | Amobarbital | 0 | 675 | - | -0.57 (-3.45 to 2.32) |
|  | Chloral hydrate | 3 | 3,261 | 1.29 (0.41-4) | 0.27 (-1.17 to 1.7) |
|  | Haloxazolam | 0 | 142 | - | -0.14 (-3.03 to 2.75) |
| N05C | Etizolam | 1 | 25,726 | 0.05 (0-0.38) | -3.27 (-5.31 to -1.23) |
|  | Alprazolam | 350 | 539,412 | 0.91 (0.81-1.01) | -0.14 (-0.29 to 0.01) |
|  | Ethyl loflazepate | 0 | 1,901 | - | -1.24 (-4.12 to 1.65) |
|  | Diazepam | 125 | 283,008 | 0.62 (0.51-0.73) | -0.69 (-0.94 to -0.42) |
|  | Lorazepam | 297 | 452,691 | 0.92 (0.82-1.03) | -0.12 (-0.28 to 0.04) |
|  | Clotiazepam | 1 | 4,635 | 0.3 (0.04-2.14) | -1.11 (-3.14 to 0.93) |
|  | Bromazepam | 6 | 42,166 | 0.2 (0.08-0.44) | -2.15 (-3.23 to -1.05) |
|  | Hydroxyzine | 63 | 132,705 | 0.67 (0.51-0.85) | -0.58 (-0.93 to -0.21) |
|  | Cloxazolam | 1 | 4,546 | 0.31 (0.04-2.19) | -1.08 (-3.12 to 0.95) |
|  | Dandospirone | 0 | 1,673 | - | -1.13 (-4.01 to 1.75) |
|  | Tofisopam | 0 | 758 | - | -0.62 (-3.51 to 2.26) |
