# Supplementary material for: An integrative approach using real-world data to identify alternative therapeutic uses of existing drugs
Source: PLoS One. 2018 Oct 9;13(10):e0204648. doi: 10.1371/journal.pone.0204648 (PMC6177143; doi:10.1371/journal.pone.0204648)
Supplement: S10 Table — Risperidone, aripiprazole, olanzapine, quetiapine, haloperidol, chlorpromazine, promethazine, prochlorperazine, paliperidone, zolpidem, eszopiclone, alprazolam, diazepam, lorazepam, and hydroxyzine were inversely associated with UC. (DOCX) [file pone.0204648.s010.docx]

S10 Table. Association between psycholeptics and ulcerative colitis (FAERS database)

Risperidone, aripiprazole, olanzapine, quetiapine, haloperidol, chlorpromazine, promethazine, prochlorperazine, paliperidone, zolpidem, eszopiclone, alprazolam, diazepam, lorazepam, and hydroxyzine were inversely associated with UC.

| ATC code | Drugs | Ulcerative colitis(PT: 10009900) | | | |
| --- | --- | --- | --- | --- | --- |
|  |  | Case | Non-cases | ROR ( Lower - Upper ) | IC ( Lower to Upper ) |
| N05A | Risperidone | 13 | 226,006 | 0.18 (0.1-0.31) | -2.38 (-3.15 to -1.6) |
|  | Aripiprazole | 14 | 140,783 | 0.31 (0.18-0.52) | -1.61 (-2.35 to -0.86) |
|  | Olanzapine | 21 | 223,445 | 0.3 (0.19-0.45) | -1.71 (-2.32 to -1.09) |
|  | Quetiapine | 36 | 361,922 | 0.31 (0.22-0.43) | -1.65 (-2.12 to -1.17) |
|  | Levomepromazine | 1 | 20,238 | 0.16 (0.02-1.1) | -1.89 (-3.93 to 0.14) |
|  | Haloperidol | 7 | 101,141 | 0.22 (0.1-0.45) | -2.05 (-3.07 to -1.03) |
|  | Chlorpromazine | 3 | 35,513 | 0.27 (0.08-0.82) | -1.62 (-3.06 to -0.17) |
|  | Blonaserin | 0 | 1,090 | - | -0.43 (-3.31 to 2.45) |
|  | Perospirone | 0 | 1,566 | - | -0.58 (-3.46 to 2.3) |
|  | Zotepine | 1 | 2,130 | 1.48 (0.2-10.48) | 0.25 (-1.78 to 2.29) |
|  | Sulpiride | 1 | 15,457 | 0.2 (0.02-1.44) | -1.56 (-3.6 to 0.47) |
|  | Prochlorperazine | 21 | 131,606 | 0.5 (0.32-0.76) | -0.96 (-1.57 to -0.34) |
|  | Paliperidone | 1 | 53,070 | 0.06 (0-0.42) | -3.16 (-5.19 to -1.11) |
|  | Bromperidol | 0 | 785 | - | -0.32 (-3.2 to 2.56) |
|  | Perphenazine | 1 | 12,661 | 0.25 (0.03-1.76) | -1.33 (-3.36 to 0.71) |
|  | Propericiazine | 1 | 1,835 | 1.71 (0.24-12.17) | 0.34 (-1.7 to 2.37) |
|  | Tiapride | 0 | 6,537 | - | -1.62 (-4.5 to 1.26) |
| N05B | Ramelteon | 0 | 14,402 | - | -2.48 (-5.36 to 0.4) |
|  | Brotizolam | 5 | 30,554 | 0.51 (0.21-1.23) | -0.84 (-2.01 to 0.34) |
|  | Zolpidem | 71 | 411,914 | 0.54 (0.42-0.68) | -0.87 (-1.21 to -0.53) |
|  | Flunitrazepam | 2 | 23,937 | 0.26 (0.06-1.05) | -1.52 (-3.18 to 0.14) |
|  | Triazolam | 4 | 25,778 | 0.49 (0.18-1.3) | -0.88 (-2.16 to 0.41) |
|  | Nitrazepam | 101 | 34,217 | 9.32 (7.66-11.32) | 3.1 (2.81 to 3.38) |
|  | Zopiclone | 18 | 88,309 | 0.64 (0.4-1.01) | -0.61 (-1.27 to 0.04) |
|  | Estazolam | 4 | 8,505 | 1.48 (0.55-3.94) | 0.43 (-0.85 to 1.72) |
|  | Rilmazafone | 1 | 3,735 | 0.84 (0.11-5.98) | -0.13 (-2.16 to 1.91) |
|  | Eszopiclone | 4 | 56,920 | 0.22 (0.08-0.58) | -1.93 (-3.22 to -0.64) |
|  | Lormetazepam | 2 | 11,871 | 0.53 (0.13-2.11) | -0.67 (-2.33 to 0.99) |
|  | Phenobarbital | 12 | 59,365 | 0.64 (0.36-1.11) | -0.61 (-1.41 to 0.18) |
|  | Quazepam | 0 | 3,031 | - | -0.97 (-3.85 to 1.91) |
|  | Triclofos | 2 | 413 | 15.23 (3.79-61.12) | 1.41 (-0.26 to 3.07) |
|  | Suvorexant | 0 | 4,417 | - | -1.27 (-4.15 to 1.62) |
|  | Flurazepam | 0 | 14,065 | - | -2.45 (-5.33 to 0.43) |
|  | Bromovalerylurea | 0 | 331 | - | -0.15 (-3.03 to 2.74) |
|  | Nimetazepam | 0 | 181 | - | -0.08 (-2.97 to 2.81) |
|  | Amobarbital | 0 | 675 | - | -0.28 (-3.16 to 2.6) |
|  | Chloral hydrate | 0 | 3,264 | - | -1.03 (-3.91 to 1.85) |
|  | Haloxazolam | 0 | 142 | - | -0.06 (-2.95 to 2.83) |
| N05C | Etizolam | 4 | 25,723 | 0.49 (0.18-1.3) | -0.88 (-2.16 to 0.41) |
|  | Alprazolam | 111 | 539,651 | 0.65 (0.53-0.77) | -0.62 (-0.89 to -0.34) |
|  | Ethyl loflazepate | 0 | 1,901 | - | -0.68 (-3.56 to 2.2) |
|  | Diazepam | 39 | 283,094 | 0.43 (0.31-0.59) | -1.19 (-1.64 to -0.72) |
|  | Lorazepam | 117 | 452,871 | 0.81 (0.67-0.97) | -0.3 (-0.56 to -0.03) |
|  | Clotiazepam | 1 | 4,635 | 0.68 (0.09-4.81) | -0.31 (-2.34 to 1.73) |
|  | Bromazepam | 13 | 42,159 | 0.97 (0.56-1.67) | -0.04 (-0.81 to 0.73) |
|  | Hydroxyzine | 23 | 132,745 | 0.54 (0.36-0.81) | -0.85 (-1.43 to -0.25) |
|  | Cloxazolam | 1 | 4,546 | 0.69 (0.09-4.91) | -0.29 (-2.33 to 1.75) |
|  | Dandospirone | 0 | 1,673 | - | -0.62 (-3.5 to 2.27) |
|  | Tofisopam | 0 | 758 | - | -0.31 (-3.19 to 2.57) |
