# Supplementary material for: An integrative approach using real-world data to identify alternative therapeutic uses of existing drugs
Source: PLoS One. 2018 Oct 9;13(10):e0204648. doi: 10.1371/journal.pone.0204648 (PMC6177143; doi:10.1371/journal.pone.0204648)
Supplement: S12 Table — Gene expression microarray data were extracted using the NextBio database for bioinformatics analysis. The NextBio database integrates raw data from the open resource GEO by a normalized ranking approach and stores processed data as datasets with a NextBio internal ID. Datasets extracted using the NextBio database are applicable for comparisons of data from different studies. The inclusion criteria for datasets in this study were as follows: 1) mRNA expression data of humans; 2) comparison of compound treatment vs a vehicle control or affected tissue from patients vs a normal control; 3) high signal-to-noise ratio. Detailed information of experimental settings for data acquisition is described. (DOCX) [file pone.0204648.s012.docx]

S12 Table. Microarray datasets for IBD and compound treatment.

|  | NextBio  Internal ID | No. of samples | Description | Platform |
| --- | --- | --- | --- | --- |
|  |  |  |  |  |
| Haloperidol | haloperidol_277 | Haloperidol (20 μM, 8hr)=2 | Comparison of gene changing in hepatocytes of female donor treated with Haloperidol and untreated group | Affymetrix GeneChip Human HG_U133 Plus 2.0 |
|  |  | Haloperidol (0 μM, 8hr)=2 |  |  |
| Diazepam | diazepam_511 | Diazepam (250 μM, 8hr)=2 | Comparison of gene changing in hepatocytes of female donor treated with Diazepam and untreated group | Affymetrix GeneChip Human HG_U133 Plus 2.0 |
|  |  | Diazepam (0 μM, 8hr)=2 |  |  |
| Hydroxyzine | hydroxyzine_341 | Hydroxyzine (40 μM, 8hr)=2 | Comparison of gene changing in hepatocytes of female donor treated with Hydroxyzine and untreated group | Affymetrix GeneChip Human HG_U133 Plus 2.0 |
|  |  | Hydroxyzine (0 μM, 8hr)=2 |  |  |
| Crohn's disease (CD) | GSE6731_2 | CD=7 | Comparison of genes changing in affected colon from Crohn's disease patient and colon from normal subject | Affymetrix GeneChip Human HG_U95A-E version [1 or 2] |
|  |  | normal= 4 |  |  |
| Ulcerative Colitis (UC) | GSE6731_4 | UC=5 | Comparison of genes changing in affected colon from Ulcerative Colitis patient and colon from normal subject | Affymetrix GeneChip Human HG_U95A-E version [1 or 2] |
|  |  | normal= 4 |  |  |
