# Supplementary material for: An integrative approach using real-world data to identify alternative therapeutic uses of existing drugs
Source: PLoS One. 2018 Oct 9;13(10):e0204648. doi: 10.1371/journal.pone.0204648 (PMC6177143; doi:10.1371/journal.pone.0204648)
Supplement: S13 Table — For each compound, the bioset generated from compound treatment together with biosets from samples acquired from patients with CD or UC were subjected to meta-analysis to identify for DEGs, which were up-regulated in IBD but down-regulated by psycholeptic treatment. DEGs, which were up-regulated in IBD listed as either up-regulated or down-regulated by tiapride, served as controls. The overall score is an internal score, calculated using the meta-analysis tool, indicates a correlation between DEGs and the analyzed biosets. DEGs with p <0.05 are listed. (DOCX) [file pone.0204648.s013.docx]

S13 Table. Differentially expressed genes (DEGs) shared between IBD and treatment with psycholeptics.

|  | Haloperidol (↓) | | Diazepam (↓) | | Hydroxyzine (↓) | | Tiapride (↓) | | Tiapride (↑) | |
| --- | --- | --- | --- | --- | --- | --- | --- | --- | --- | --- |
|  | Gene | Overall score | Gene | Overall score | Gene | Overall score | Gene | Overall score | Gene | Overall score |
| IBD (↑) | G0S2 | 283.3 | CXCL2 | 295.2 | CXCL2 | 291.9 | HLA-DMB | 245 | UBD | 273.2 |
|  | BCL2A1 | 271.3 | IL8 | 284.9 | G0S2 | 269.5 | NUP210 | 229.1 | HLA-DRA | 240.8 |
|  | BIRC3 | 243.5 | IL1RN | 279.4 | BIRC3 | 263.4 | CTSH | 224.5 | CRK | 223.9 |
|  | GBP1 | 242 | BIRC3 | 265.9 | GBP1 | 257.2 | TAPBP | 220.3 | LPIN1 | 215.2 |
|  | FSCN1 | 222.3 | C4BPB | 246.2 | IER3 | 235 | FCN1 | 219 | CASP10 | 193.1 |
|  | HLA-DPA1 | 209 | CEBPD | 232.4 | HYAL1 | 206.2 | ITGB4 | 200.9 | ANGPTL2 | 191.1 |
|  | SP110 | 202.7 | GBP1 | 225.2 | HSPA6 | 204.1 | NOP56 | 196.9 | PIK3R3 | 185.6 |
|  | AIF1 | 193.6 | KYNU | 220.7 | NOP56 | 191.6 | RBPMS | 185.9 | BLVRA | 181.3 |
|  | SH3GL1 | 190.8 | STOM | 213.6 | SRF | 179 | ITGAL | 180.6 | KPNA6 | 164.8 |
|  | ITGA6 | 181.9 | PIK3R3 | 212.2 | RSL1D1 | 156.5 | ALDH4A1 | 180.6 | LIG3 | 162.9 |
|  | IL16 | 181.9 | NUP210 | 206.7 |  |  | ABHD14A | 161.4 |  |  |
|  |  |  | SERPINH1 | 185.7 |  |  |  |  |  |  |
|  |  |  | ITGA5 | 185.3 |  |  |  |  |  |  |
|  |  |  | INPP1 | 180.9 |  |  |  |  |  |  |
|  |  |  | RBPMS | 176.1 |  |  |  |  |  |  |
|  |  |  | CFLAR | 170.6 |  |  |  |  |  |  |
|  |  |  | HCP5 | 168.5 |  |  |  |  |  |  |
|  |  |  | NOP16 | 152.6 |  |  |  |  |  |  |
|  |  |  | NUP214 | 150.6 |  |  |  |  |  |  |
|  |  |  | OSMR | 140.4 |  |  |  |  |  |  |
